# The burden of diseases, injuries, and risk factors by voivodship in Poland, 1990–2023: a systematic analysis for the Global Burden of Disease Study 2023

**DOI:** 10.1016/j.lanepe.2025.101431

**Published:** 2025-09-03

**Authors:** GBD 2023 Poland Collaborators, Roman Topor-Madry, Anna Bilik, Zbigniew J. Król, Maciej Miłkowski, Adam Maciejczyk, Kamila Malinowska, Agnieszka Micek, Ayman Ahmed, Mohammed Ahmed Akkaif, Abid Ali, Waad Ali, Mohammed A. Alsabri, Mohammad Al-Wardat, Razman Arabzadeh Bahri, Shahkaar Aziz, Maciej Banach, Mohammad-Mahdi Bastan, Maryam Beiranvand, Sonu Bhaskar, Catherine Bisignano, Vijay Kumar Chattu, Sheng-Chia Chung, Nicole Davis Weaver, Kuldeep Dhama, Arkadiusz Marian Dziedzic, Temitope Cyrus Ekundayo, Chadi Eltaha, Patrick Fazeli, Nuno Ferreira, Xiang Gao, Miglas Welay Gebregergis, Scott D. Glenn, Snigdha Gulati, Simon I. Hay, Chengxi Hu, M Azhar Hussain, Jacek Jerzy Jozwiak, Adnan Kisa, Munjae Lee, Johan Månsson, Hadush Negash Meles, Tomasz Miazgowski, Irmina Maria Michalek, Ali H. Mokdad, Christopher J.L. Murray, Mohsen Naghavi, Van Thanh Nguyen, Fred Nugen, Michal Ordak, Samuel M. Ostroff, Mahesh P. A, Maja Pasovic, Shakthi Kumaran Ramasamy, Elrashdy Redwan, Cameron John Sabet, Mohammad Reza Saeb, Mehdi Safari, Jennifer Saulam, Mohammad Ali Shamshirgaran, Mahabalesh Shetty, Aminu Shittu, Emmanuel Edwar Siddig, Chandan Kumar Swain, Lukasz Szarpak, Muhammad Waqas, Marcin W. Wojewodzic, Bogdan Wojtyniak

**Keywords:** Poland, Europe, Voivodship, Life expectancy, Decomposition, Disability-adjusted life-years DALYs, Risk factors, GBD 2023, Population health

## Abstract

**Background:**

Since 1990, major political and economic transformations in Poland have substantially impacted the country's health systems and shaped its health policies. We aimed to better understand the changes in population health in Poland by location and demographic groups, using data from the Global Burden of Diseases, Injuries, and Risk Factors Study (GBD) 2023.

**Methods:**

GBD 2023 evaluates outcomes for 375 diseases and injuries, 292 causes of death, and 88 risk factors in 204 countries and territories. We analysed mortality, life expectancy, and risk-attributable burden, years lived with disability (YLDs), years of life lost due to premature death (YLLs), and disability-adjusted life-years (DALYs; overall burden as a sum of years of lost life due to premature death and years lived with disability), and included a decomposition of change in life expectancy by cause and location in Poland and its regions (voivodships) between 1990 and 2023 on the background of selected European countries. Estimates were produced for Poland at the national and subnational level by age (25 age groups), sex (males, females, and all sexes combined), location, and year. Final point estimates are reported with 95% uncertainty intervals (UIs) representing the 2.5th and 97.5th percentile of the distribution from 250 draws for each metric.

**Findings:**

Between 1990 and 2023, life expectancy in Poland for male and female sexes combined increased from 71.2 (95% UI 71.1–71.3) to 78.6 (78.5–78.7) years. Male life expectancy increased more than female during this period; male life expectancy increased from 66.8 (66.7–66.9) to 74.9 (74.8–75.0), while female life expectancy increased from 75.7 (75.6–75.8) to 82.2 (82.1–82.3). Reductions in deaths from ischaemic heart disease and stroke drove most improvements; in 1990, there were an estimated 109,000 (104,000–115,000) and 68,300 (63,700–73,600) deaths due to IHD and stroke, respectively, and in 2023 there were 85,400 (76,800–90,800) and 39,500 (35,000–42,900). The voivodship with the highest age-standardised DALY rates per 100,000 in 2023 was Łódzkie with 25,607 (22,602–29,217), while the lowest was Małopolskie with 22,113 (19,290–25,418). Nationally, age-standardised DALYs declined 33.9% (30.7–36.8) from 1990 to 2023. Smoking and high blood pressure were the leading risk factors throughout the period of study, while alcohol use showed the greatest increase in DALY rates between 1990 and 2023 with 35.2% (3.5–65.1). Risk-attributable age-standardised DALYs rates declined for high blood pressure (1990: 5723.8 [4759.8–6578.3]; 2023: 2053.7 [1657.2–2362.4]) and high BMI (1990: 2226.4 [966.8–3512.2]; 2023: 1923.4 [839.0–2907.8]).

**Interpretation:**

Since 1990, life expectancy has increased in Poland for both males and females, with males gaining more years of expected life. In 2023 it was higher than in eastern and central Europe but behind western Europe. Results from GBD 2023 highlight the disparities in the burden of disease across voivodships and suggest that risk-attributable burden in Poland, at both the national and voivodship levels, can be addressed by focusing on several modifiable risk factors, especially smoking and alcohol use.

**Funding:**

10.13039/100000865Gates Foundation.

**Research in context**

**Evidence before this study**

The Global Burden of Diseases, Injuries, and Risk Factors Study (GBD) has provided regular updates on complex patterns and trends in population health for the past three decades. In Poland, metrics presented in GBD, such as disability-adjusted life-years (DALY), have been used to design policy decision frameworks and guide decision making since 2019. However, despite the overall value and use of GBD in policy making at the national and regional levels in Poland, there has not been, to the best of our knowledge, a comprehensive analysis of disease burden based on the study. According to the systematic search performed for the years 1990–2023 on 8 September 2023, 133 publications appeared in OVID, EMBASE, MEDLINE, and Cochrane Library databases that showed GBD indicators (DALYs, years lived with disability [YLDs], years of life lost to premature mortality [YLLs], and life expectancy) specific for Poland. The search strategy, along with a flowchart outlining the excluded and included studies, can be found in the appendix (Appendix 1, Section 1).

**Added value of this study**

This paper analyses GBD estimates providing a three-decade perspective of morbidity, mortality, and risk-attributable burden for Poland at both national and subnational levels and compares these results with central, eastern, and western European countries. This is the first time that GBD estimates for Poland across all voivodships have been analysed in a publication.

**Implications of all the available evidence**

Our study contributes detailed insights on mortality and morbidity in Poland, as well as quantifications of risk-attributable burden, through a variety of population health metrics. These comprehensive estimates are vital for Polish policy makers and stakeholders to evaluate health needs, tailor interventions, and distribute resources; indeed, evidence-based strategies are foundational for the design and implementation of effective health policy programmes and provide a benchmark on Poland's health system strategy.

## Introduction

Poland, the most populous country in central Europe and Europe's eighth largest,^1^ had a population of 38 million in 2023, with 16 administrative regions called “voivodships”.^2^, ^3^, ^4^ Poland's population health indicators are comparable to those of other central European countries, such as Czechia and Hungary, lag behind western Europe, but are ahead of eastern European countries.^5^, ^6^, ^7^, ^8^ Poland is facing an ageing population pattern, a limited and ageing cadre of medical professionals, unmet health-care needs, an unfavourable risk factors profile, and moderate health care expenditure.^9^ In health care, Poland has transitioned from the centrally funded, single-payer Semashko model of health care, free to everyone, to more decentralised and partially privatised systems.^10^, ^11^, ^12^ These trends track broadly to major social, political, and economic transitions in Poland since 1990, as Poland and other central and eastern European countries shifted from Soviet-style communism to free-market liberalism. In 1990, Polish men could expect to live about 66 years, while women's life expectancy was around 75 years. With the transition to a market economy and health-care system reforms, life expectancy began to rise. Health expenditure gradually increased, enabling the modernisation of medical infrastructure and the introduction of advanced treatments.^9^^,^^13^ Non-communicable diseases (NCDs) remained a major public health challenge; however, mortality from cardiovascular diseases started to decline in the late 1990s and continued to decrease throughout the 2000s. Smoking cessation efforts played a crucial role in reducing cardiovascular mortality, though lung cancer, particularly in men, remained a significant issue during this period.^13^, ^14^, ^15^

Regional disparities, however, remain a challenge. These regional differences have been influenced by economic disparities, lifestyle differences, health-care infrastructure, and availability of health-care professionals. Urban areas typically had better access to specialised care and more advanced medical technologies, while rural areas faced longer wait times and limited access to health care.^13^^,^^16^, ^17^, ^18^, ^19^, ^20^ Most of the health information in Poland comes from vital statistics, claims data, several specific registries (ie, national oncology registry, communicable disease registry) and several main national population surveys,^21^, ^22^, ^23^, ^24^ with lack of a continuous system of monitoring morbidity and risk factors in key areas.

For over 30 years, the Global Burden of Diseases, Injuries, and Risk Factors Study (GBD) has delivered timely, comprehensive estimates of health and health loss to enable evidence-based decision making and informed the allocation of resources to improve population health.^25^ This paper examines health trends in Poland and its regions from 1990 to 2023, including estimates of life expectancy and disability-adjusted life-years (DALYs), for fatal and non-fatal diseases and injuries, as well as risk factors in the context of 12 central European countries, and average value for the western, central, and eastern European regions and widens and explores the most recent estimates from GBD 2023 analyses.^25^, ^26^, ^27^, ^28^, ^29^ Starting from the Polish translation of the GBD handbook in 2000,^30^ presentation of the methodology of calculating DALYs in the handbook of health care needs assessment,^31^ and previous editions of GBD publications,^27^^,^^29^^,^^32^, ^33^, ^34^ this is the first time comprehensive estimates for Poland at the national and subnational levels across 30 years have been presented. This manuscript was produced as part of the GBD Collaborator Network and in accordance with the GBD Protocol.^35^

## Methods

### Overview

GBD 2023, the latest iteration, produced estimates for 375 diseases and injuries, 288 causes of death, and 88 risk factors by age-sex-location-year for 25 age groups from birth to 95 years and older; for males, females, and all sexes combined; in 204 countries and territories, organised into 21 regions and seven super-regions; and for each year from 1990 to 2023. GBD 2023 also has subnational analyses for 20 countries and territories, including Poland. Fatal and non-fatal causes were grouped into four nested cause categories. The broadest category, Level 1, comprises three groups: communicable, maternal, neonatal, and nutritional (CMNN) diseases; non-communicable diseases (NCDs); and injuries. Levels 2–4 in the GBD cause hierarchy are disaggregated and become increasingly specific. The measures of burden are expressed in counts and age-standardised rates per 100,000 person-years. Uncertainty intervals (UIs) were calculated using the 2.5th and 97.5th percentiles based on the tail-end distribution of 250 draws. GBD follows the Guidelines for Accurate and Transparent Health Estimates Reporting (GATHER) statement. A completed GATHER checklist is included with the Supplementary Materials (Appendix 1, Section 2, Table S4). A detailed description of the measures and methods used in GBD 2023 has been published in the past.^27^^,^^29^^,^^36^^,^^37^

### Data sources and processing

Demographic data on the population of Poland by sex and by five-year age groups in different periods were prepared and incorporated into population estimates at the national and subnational levels. Data were obtained from statistical yearbooks and censuses for available years (1946, 1950, 1955, 1978, 1988) and for the entire period 1990–2023 and mapped to division into 16 voivodships (since 1999). A map of Poland showing voivodship names and estimated populations for 2023 is included in the Appendix (Section 1, Figure S13). Morbidity was based on claims data and age-sex-location-year and total and cause-specific mortality data for 1974–2023 from the vital statistics system in Poland. All data sources used for estimation of diseases, injuries, and risk factors in Poland are available online via the Global Health Data Exchange: https://ghdx.healthdata.org/geography/poland. GBD 2023 used the following software: Python version 3.10.4, Stata version 13.1, and R version 4.2.1.

### Estimating the fatal and non-fatal burden of diseases and injuries

Age-specific mortality rates and standard demographic methods were used to calculate life expectancy.^28^ The contributions of specific causes to changes in life expectancy over the study period are calculated by decomposing differences in life expectancy by age, then further decomposing by cause-age, and finally by aggregating cause-age-specific contributions across age groups to produce cause-specific contributions.^38^ Cause of Death Ensemble modelling (CODEm) is used in GBD 2023 to estimate the causes of death by age, sex, location, and time period.^39^ CODEm was used to generate final mortality estimates. Estimates are adjusted for assumed independent comorbidity.

Estimates of incidence, prevalence, YLLs (number of years of life lost due to premature death), YLDs (the number of years lived disabled weighted by level of disability), and DALYs (quantifies overall burden as a sum of years of lost life due to premature death and years lived with disability) were produced for Poland at the national and subnational level for GBD 2023. For most causes, prevalence and incidence were modelled using DisMod-MR 2.1, a Bayesian meta-regression disease modelling tool; a new tool for GBD 2023, DisMod-AT, was used to model four causes (type 1 diabetes, major depressive disorder, anxiety disorders, and autism spectrum disorders). Data with known biases, such as alternative case definitions or measurement methods, were adjusted using a tool known as MR-BRT (meta-regression—Bayesian, regularised, trimmed). YLL estimates were calculated by multiplying age group mortality estimates by life expectancy derived from GBD estimates. YLDs were calculated based on estimates of the number of YLDs per sequela for specific causes and incorporate analyses of disability weights. Disability weights range from 0 (a state of full health) to 1 (death) and are based on community surveys of cause-specific health loss in the general population.^40^^,^^41^ DALYs equal the sum of YLLs and YLDs.

### Estimating the disease burden attributable to risk factors

GBD 2023 estimated the risk-attributable burden of 88 risk factors. Risk factor estimation was based on a comparative risk assessment framework for modifiable risk factors, organised in a four-level hierarchy. Four inputs were combined for the risk-factor estimation process: 1) exposure; 2) relative risk; 3) theoretical minimum risk exposure level (TMREL); and 4) mortality and morbidity for each risk–outcome. For each risk factor–outcome pair, meta-analyses of published literature were performed to estimate the relative risk (RR) of non-fatal health loss, mortality, or both. Second, by location, sex, and age group, MR-BRT was used to adjust for bias in risk factor exposure data and perform age-sex splitting. The distribution of risk factor exposure by age, sex, and location was then estimated. Finally, the attributable burden of disease is estimated by comparing the burden due to the current distribution of risk factors with the hypothetical burden due to the distribution of the theoretical minimum level of risk exposure.^26^

### Role of the funding source

The funders of the study had no role in study design, data collection, data analysis, data interpretation, or the writing of the report.

## Results

### Life expectancy in Poland and subnational units in the period 1990 to 2023

In Poland from 1990 to 2023, life expectancy for males and females combined increased from 71.2 years (95% UI 71.1–71.3) to 78.6 years (78.5–78.7), dropping to 75.7 years (75.7–75.8) in 2021. It exceeded averages for central and eastern Europe but fell short of western Europe (76.3 years [76.3–76.3] in 1990 versus 82.1 years [82.1–82.1] in 2023). The only countries in central Europe with higher life expectancy than Poland were Slovenia, Czechia, and Albania (Croatia had the same level as Poland), all of which had also greater improvements from 1990. The increase of life expectancy from 1990 to 2023 for males was bigger than for females; this was similar in all analysed countries but very different across Polish regions: from the biggest gain, 9.3 years in males in Pomorskie, to the smallest, 4.4 years in females in Świętokrzyskie. Regional disparities persisted: Podkarpackie and Małopolskie led with 80.0 years (79.5–80.6 and 79.5–80.4, respectively), while Łódzkie was lowest at 77.6 years (77.1–78.1). A sex gap in life expectancy existed across regions in 2023, with Małopolskie having the highest life expectancy for males at 76.5 years (75.9–77.1), Podkarpackie and Podlaskie for females (83.7 [82.8–83.8 and 82.9–84.5, respectively]), and Śląskie the lowest for females (81.3 years [80.8–81.8]) and Łódzkie the lowest for males (73.6 years [72.8–74.3]) (Table 1).

**Table 1:** Life expectancy at birth by sex, western, central, and eastern Europe and Poland's 16 voivodships in 1990, 2021 and 2023.

Life expectancy at birth, 1990, 2021, 2023									
Location	1990			2021			2023		
	Both	Male	Female	Both	Male	Female	Both	Male	Female
**Eastern Europe**	69.7 (69.7–69.8)	64.5 (64.5–64.6)	74.5 (74.5–74.6)	71.0 (70.9–71.0)	66.6 (66.5–66.6)	75.2 (75.2–75.3)	73.3 (72.7–73.6)	67.8 (67.1–68.4)	78.6 (78.3–78.9)
**Western Europe**	76.3 (76.3–76.3)	73.0 (72.9–73.0)	79.6 (79.5–79.6)	81.7 (81.7–81.7)	79.3 (79.3–79.3)	84.1 (84.1–84.1)	82.1 (82.1–82.1)	79.7 (79.7–79.7)	84.4 (84.4–84.4)
**Central Europe**	70.8 (70.8–70.9)	67.1 (67.0–67.2)	74.7 (74.6–74.7)	74.8 (74.8–74.8)	71.4 (71.4–71.4)	78.3 (78.3–78.3)	77.7 (77.6–77.8)	74.4 (74.3–74.5)	81.0 (80.9–81.1)
Albania	71.9 (71.5–72.3)	69.2 (68.7–69.7)	75.0 (74.5–75.5)	76.9 (76.7–77.1)	74.5 (74.2–74.7)	79.5 (79.3–79.7)	79.6 (79.2–80.1)	77.3 (76.6–77.9)	82.0 (81.4–82.6)
Bosnia and Herzegovina	72.4 (72.2–72.6)	69.5 (69.2–69.8)	75.3 (75.0–75.6)	74.6 (74.5–74.7)	72.0 (71.9–72.2)	77.2 (77.1–77.3)	77.7 (77.2–78.2)	75.3 (74.6–76.0)	80.1 (79.4–80.8)
Bulgaria	71.2 (71.1–71.4)	68.0 (67.8–68.2)	74.7 (74.5–74.9)	72.2 (72.2–72.3)	68.9 (68.8–69.0)	75.8 (75.7–75.9)	75.9 (75.7–76.1)	72.5 (72.2–72.7)	79.5 (79.3–79.7)
Croatia	72.5 (72.4–72.6)	68.7 (68.5–68.9)	76.2 (76.0–76.3)	77.0 (76.9–77.1)	73.8 (73.7–74.0)	80.1 (80.0–80.3)	78.6 (78.4–78.8)	75.5 (75.2–75.8)	81.7 (81.5–81.9)
Czechia	71.5 (71.4–71.6)	67.7 (67.6–67.9)	75.4 (75.3–75.5)	77.4 (77.3–77.4)	74.2 (74.2–74.3)	80.6 (80.6–80.7)	79.8 (79.7–79.9)	76.9 (76.7–77.1)	82.7 (82.6–82.9)
Hungary	69.4 (69.3–69.5)	65.2 (65.0–65.4)	73.8 (73.7–73.9)	74.3 (74.2–74.3)	70.8 (70.7–70.9)	77.8 (77.7–77.8)	76.8 (76.7–77.0)	73.4 (73.2–73.6)	80.1 (79.9–80.2)
North Macedonia	71.1 (71.0–71.3)	69.0 (68.8–69.3)	73.4 (73.1–73.6)	73.3 (73.2–73.4)	71.2 (71.1–71.4)	75.4 (75.3–75.6)	77.0 (76.7–77.4)	75.2 (74.6–75.7)	79.0 (78.5–79.4)
Montenegro	73.1 (72.9–73.3)	70.2 (70.0–70.5)	75.9 (75.7–76.2)	73.2 (73.1–73.4)	70.5 (70.3–70.7)	76.3 (76.1–76.5)	77.2 (76.9–77.4)	74.9 (74.5–75.3)	79.6 (79.2–79.9)
**Poland**	**71.2 (71.1–71.3)**	**66.8 (66.7–66.9)**	**75.7 (75.6–75.8)**	**75.7 (75.7–75.8)**	**71.9 (71.8–71.9)**	**79.7 (79.7–79.7)**	**78.6 (78.5–78.7)**	**74.9 (74.8–75.1)**	**82.2 (82.1–82.3)**
*Dolnośląskie*	70.8 (70.4–71.1)	66.3 (65.9–66.8)	75.3 (74.9–75.8)	75.6 (75.4–75.8)	71.7 (71.4–71.9)	79.6 (79.4–79.8)	78.4 (77.9–78.9)	74.6 (74.0–75.3)	82.0 (81.5–82.6)
*Kujawsko-Pomorskie*	71.1 (70.7–71.5)	66.7 (66.1–67.2)	75.6 (75.1–76.1)	75.2 (75.0–75.4)	71.5 (71.2–71.8)	79.1 (78.8–79.3)	78.1 (77.6–78.6)	74.5 (73.8–75.2)	81.7 (81.0–82.4)
*Łódzkie*	70.6 (70.3–71.0)	66.1 (65.6–66.6)	75.3 (74.8–75.7)	74.8 (74.6–75.0)	70.7 (70.4–71.0)	79.0 (78.8–79.2)	77.6 (77.1–78.1)	73.6 (72.8–74.3)	81.6 (81.0–82.1)
*Lubelskie*	72.1 (71.7–72.4)	67.6 (67.1–68.1)	76.8 (76.4–77.3)	75.4 (75.2–75.6)	71.4 (71.1–71.7)	79.6 (79.3–79.8)	78.6 (78.2–79.1)	74.5 (73.8–75.3)	82.7 (82.2–83.4)
*Lubuskie*	70.6 (70.2–71.1)	66.1 (65.5–66.8)	75.4 (74.8–75.9)	74.8 (74.5–75.0)	70.8 (70.4–71.1)	79.0 (78.7–79.4)	78.0 (77.4–78.6)	74.2 (73.3–75.1)	81.8 (81.0–82.5)
*Małopolskie*	72.6 (72.2–72.9)	68.4 (67.9–68.9)	76.8 (76.4–77.2)	77.3 (77.1–77.4)	73.6 (73.3–73.8)	81.1 (80.9–81.3)	80.0 (79.5–80.4)	76.5 (75.9–77.1)	83.3 (82.8–83.8)
*Mazowieckie*	70.5 (70.2–70.7)	66.1 (65.7–66.5)	75.0 (74.7–75.3)	75.9 (75.8–76.1)	71.9 (71.7–72.1)	80.0 (79.8–80.2)	78.7 (78.4–79.1)	75.1 (74.5–75.6)	82.3 (81.8–82.7)
*Opolskie*	71.7 (71.3–72.1)	67.3 (66.7–67.8)	76.1 (75.5–76.6)	76.1 (75.9–76.4)	72.4 (72.0–72.7)	80.1 (79.7–80.4)	78.9 (78.3–79.6)	75.4 (74.5–76.2)	82.5 (81.7–83.3)
*Podkarpackie*	72.7 (72.4–73.1)	68.6 (68.1–69.1)	76.9 (76.5–77.4)	76.7 (76.5–76.9)	72.9 (72.6–73.2)	80.7 (80.4–80.9)	80.0 (79.5–80.6)	76.3 (75.5–77.1)	83.7 (83.1–84.4)
*Podlaskie*	72.9 (72.5–73.4)	68.5 (67.9–69.2)	77.6 (77.1–78.1)	75.9 (75.6–76.1)	71.6 (71.3–72.0)	80.5 (80.1–80.8)	79.4 (78.8–80.0)	75.2 (74.3–76.1)	83.7 (82.9–84.5)
*Pomorskie*	70.5 (70.2–70.9)	66.2 (65.7–66.8)	75.0 (74.5–75.4)	76.4 (76.2–76.6)	72.8 (72.5–73.1)	80.1 (79.9–80.4)	78.9 (78.4–79.4)	75.5 (74.9–76.3)	82.2 (81.5–82.8)
*Śląskie*	70.1 (69.9–70.4)	65.8 (65.4–66.2)	74.5 (74.1–74.8)	74.9 (74.7–75.0)	71.2 (71.0–71.4)	78.7 (78.6–78.9)	77.9 (77.5–78.3)	74.5 (73.9–75.1)	81.3 (80.8–81.8)
*Świętokrzyskie*	73.6 (73.1–73.9)	69.2 (68.6–69.8)	78.2 (77.8–78.7)	75.6 (75.3–75.8)	71.5 (71.2–71.9)	80.0 (79.7–80.3)	78.4 (77.8–78.9)	74.3 (73.4–75.1)	82.6 (81.9–83.4)
*Warmińsko-Mazurskie*	70.5 (70.0–70.9)	65.7 (65.1–66.3)	75.7 (75.2–76.2)	75.0 (74.8–75.3)	70.9 (70.5–71.2)	79.5 (79.3–79.8)	78.1 (77.6–78.7)	74.2 (73.4–75.1)	82.1 (81.4–82.8)
*Wielkopolskie*	70.9 (70.6–71.2)	66.5 (66.1–67.0)	75.5 (75.1–75.9)	76.1 (75.9–76.2)	72.4 (72.2–72.6)	79.8 (79.6–80.0)	78.6 (78.2–79.0)	75.1 (74.5–75.8)	82.0 (81.4–82.5)
*Zachodniopomorskie*	70.9 (70.5–71.3)	66.4 (65.8–67.0)	75.7 (75.2–76.2)	75.4 (75.2–75.6)	71.6 (71.3–71.9)	79.3 (79.0–79.6)	78.1 (77.6–78.7)	74.5 (73.7–75.2)	81.8 (81.2–82.6)
Romania	70.1 (70.0–70.2)	66.9 (66.8–67.1)	73.3 (73.2–73.5)	73.0 (73.0–73.0)	69.4 (69.4–69.5)	76.8 (76.8–76.9)	76.3 (76.1–76.4)	72.5 (72.3–72.7)	80.1 (80.0–80.3)
Serbia	70.1 (69.8–70.3)	67.2 (66.9–67.5)	73.0 (72.7–73.3)	73.3 (73.2–73.4)	71.0 (70.9–71.1)	75.7 (75.6–75.9)	76.0 (75.4–76.7)	73.9 (73.0–74.9)	78.2 (77.4–79.0)
Slovakia	71.0 (70.8–71.1)	66.7 (66.5–66.9)	75.5 (75.3–75.6)	74.6 (74.6–74.7)	71.2 (71.1–71.3)	78.2 (78.1–78.3)	78.2 (78.0–78.3)	74.8 (74.6–75.0)	81.5 (81.2–81.7)
Slovenia	73.3 (73.1–73.4)	69.1 (68.9–69.4)	77.2 (77.1–77.4)	80.6 (80.5–80.6)	77.6 (77.5–77.7)	83.6 (83.5–83.7)	81.7 (81.5–81.9)	79.1 (78.8–79.3)	84.4 (84.2–84.6)

**Fig. 1:**
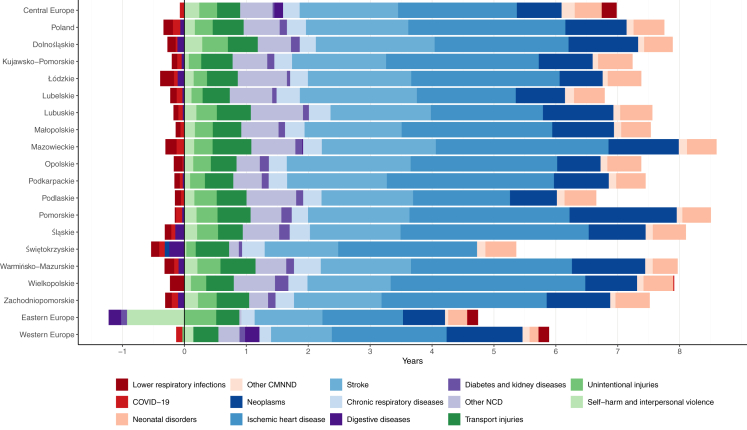
**Life expectancy decomposition by causes in Poland, 16 voivodships and European regions between 1990 and 2023.** CMMND = communicable, maternal, neonatal, and nutritional diseases. NCDs = non-communicable diseases.

The biggest increase in life expectancy in Poland between 1990 and 2023 was achieved through improvements to prevention and treatment programmes that led to decreases in IHD deaths, contributing an increase of 2.5 years nationally (highest: Wielkopolskie, 3.1 years; lowest: Podlaskie, 1.6 years); stroke, a 1.6-year improvement (highest: Opolskie, 2.0 years; lowest: Świętokrzyskie, 1.2 years); neoplasms, a 1.0-year change (highest increase: Pomorskie, 1.7; decrease: Świętokrzyskie, −0.07) (Fig. 1; Appendix 1, Section 1, Table S3). The greatest increase in life expectancy due to changes in mortality from IHD, stroke, neonatal disorders, and unintentional injuries occurred in 1995–2000; chronic respiratory and transport injuries in 1990–1995; and self-harm and interpersonal violence, as well as diabetes and kidney disease in 2010–2015. Losses in life expectancy were observed because of increases in mortality due to diabetes and kidney disease in 2015–2020 nationally (and in several voivodships in 2005–2010) and self-harm and interpersonal violence in 1990–1995, unintentional injuries 2020–2023, and IHD only locally in Lubelskie, Pomorskie, Świętokrzyskie in 2015–2020 and in Dolnośląskie and Lubuskie in 2020–2023 (Appendix 1, Section 1, Figures S1–S9).

In the analysis for the period 1990–2021, COVID-19 deaths contributed to 2 years of lost life expectancy in Poland (compared to 1.0 year in western Europe, 2.2 in central Europe, and 2.6 in eastern Europe). The magnitude of change varied by voivodship, with the largest impact in Podlaskie (decrease of 2.5 years), and the lowest in Małopolskie and Dolnośląskie (decrease of 1.7 years) (Appendix 1, Section 1, Figure S10).

### DALYs and deaths rate in Poland and its 16 voivodships

#### Improvements in health resulting in all-cause DALYs reduction from 1990

In Poland, age-standardised DALYs per 100,000 population dropped by 33.9% (95% UI 30.7–36.8) from 1990 to 2023 (1990: 35,958.8 [33,228.8–39 356.9] versus 2023: 23,774.8 [21,098.0–27 166.4]). Between 1990 and 2023, the largest regional drop was in Mazowieckie, a 37.6% decline (34.2–41.1), and the lowest was in Świętokrzyskie, a 24.9% decline (21.2–28.8). Among 13 central European countries, Poland ranked fifth in age-standardised DALY rate reduction, with Albania leading with a 38.6% decline (34.9–42.1). By contrast, the decline in western Europe was 23.3% (20.0–26.8). The absolute age-standardised DALYs were 36% higher in Poland than in western Europe in 1990 and 17% higher in 2023 (Table 2).

**Table 2:** Age-standardised all-cause DALYs per 100,000 people in western, eastern, and central European countries and subnational Poland in 1990, 2021, and 2023, with relative percentage change of the age-standardised DALYs rate.

Location	1990	2021	2023	Change % 1990–2023
**Eastern Europe**	**39,382.3 (36,576.3–42,633.5)**	**36,253.1 (33,464.4–39,616.2)**	**32,927.8 (29,875.5–36,275.3)**	**−16.4 (−19.3 to −13.4)**
**Western Europe**	**26,508.0 (23,917.3–29,394.6)**	**20,474.7 (17,776.3–23,739.3)**	**20,328.8 (17,574.4–23,628.9)**	**−23.3 (−26.8 to −20.0)**
**Central Europe**	**36,739.5 (34,068.9–39,981.3)**	**28,678.4 (26,047.1–31,992.3)**	**24,582.4 (21,942.1–27,948.4)**	**−33.1 (−35.9 to −30.3)**
Albania	36,472.2 (33,724.5–39,994.2)	26,197.6 (23,457.7–29,459.4)	22,381.1 (19,724.4–25,454.5)	−38.6 (−42.1 to **−**34.9)
Bosnia and Herzegovina	33,112.4 (30,492.2–36,401.0)	29,306.9 (26,533.8–32,825.5)	24,673.7 (21,984.8–28,052.4)	−25.5 (−29.3 to **−**21.9)
Bulgaria	35,697.1 (33,093.0–38,824.8)	33,301.9 (30,663.0–36,743.8)	27,167.3 (24,554.9–30,561.1)	−23.9 (−26.4 to **−**21.1)
Croatia	32,912.2 (30,295.8–36,005.8)	25,589.0 (22,887.4–28,751.7)	23,478.6 (20,784.3–26,621.7)	−28.7 (−31.8 to **−**25.3)
Czechia	35,011.0 (32,245.3–38,163.6)	25,193.1 (22,475.2–28,484.1)	22,138.9 (19,388.1–25,598.9)	−36.8 (−40.1 to **−**33.4)
Hungary	39,751.8 (36,850.4–43,219.1)	29,339.6 (26,741.8–32,744.8)	25,664.1 (22,974.6–28,979.2)	−35.4 (−38.1 to **−**32.6)
North Macedonia	36,417.0 (33,744.2–39,453.7)	31,617.2 (28,990.9–35,023.8)	25,104.3 (22,460.5–28,123.3)	−31.1 (−34.4 to **−**27.9)
Montenegro	31,790.2 (29,400.7–34,683.7)	31,395.5 (28,645.9–34,643.2)	25,037.4 (22,457.4–28,418.8)	−21.2 (−24.2 to **−**17.6)
Romania	38,777.1 (36,083.5–41,957.0)	31,521.8 (28,963.7–34,820.8)	26,537.5 (23,837.8–29,780.5)	−31.6 (−34.3 to **−**28.9)
Serbia	38,396.9 (35,806.8–41,401.8)	31,343.0 (28,822.3–34,512.9)	26,563.2 (23,513.3–30,036.1)	−30.8 (−34.9 to **−**26.9)
Slovakia	35,778.9 (33,082.5–39,080.3)	28,698.7 (26,049.3–31,945.7)	24,091.0 (21,501.3–27,337.8)	−32.7 (−35.6 to **−**29.7)
Slovenia	31,585.4 (28,864.9–34,844.9)	21,014.7 (18,433.9–24,286.9)	20,097.4 (17,280.0–23,403.8)	−36.4 (−40.1 to **−**32.7)
**Poland**	**35,958.8 (33,228.8–39,356.9)**	**27,360.9 (24,710.7–30,714.8)**	**23,774.8 (21,098.0–27,166.4)**	**−33.9 (−36.8 to −30.7)**
Dolnośląskie	36,613.3 (33,579.6–40,090.7)	27,498.2 (24,931.1–30,709.8)	23,982.3 (21,200.0–27,525.8)	−34.5 (−38.1 to **−**30.9)
Kujawsko-Pomorskie	36,009.9 (33,262.0–39,629.7)	27,857.2 (25,168.2–31,222.4)	24,225.8 (21,514.6–27,590.9)	−32.7 (−36.2 to **−**29.4)
Łódzkie	37,311.9 (34,292.6–40,737.3)	29,356.0 (26,425.6–32,821.5)	25,607.9 (22,602.6–29,217.5)	−31.4 (−35.2 to **−**27.6)
Lubelskie	34,358.8 (31,457.8–37,987.5)	27,903.7 (25,215.9–31,226.9)	23,813.8 (21,057.0–27,165.1)	−30.7 (−34.3 to **−**26.6)
Lubuskie	36,745.5 (33,993.4–40,358.3)	28,511.3 (25,936.0–31,757.0)	24,236.9 (21,497.1–27,841.2)	−34.0 (−38.2 to **−**30.3)
Małopolskie	33,210.6 (30,463.4–36,535.2)	25,134.9 (22,436.8–28,516.7)	22,113.6 (19,290.7–25,418.2)	−33.4 (−37.1 to **−**29.6)
Mazowieckie	37,943.7 (35,171.9–41,345.7)	27,226.3 (24,500.3–30,642.2)	23,692.7 (20,840.5–26,991.9)	−37.6 (−41.1 to **−**34.2)
Opolskie	34,950.7 (32,016.0–38,695.2)	26,814.7 (24,065.7–30,044.2)	23,390.3 (20,817.6–26,848.8)	−33.1 (−37.0 to **−**29.8)
Podkarpackie	33,038.0 (30,329.7–36,446.9)	26,050.3 (23,383.5–29,415.6)	22,290.1 (19,644.8–25,502.0)	−32.5 (−36.5 to **−**28.8)
Podlaskie	33,608.4 (30,830.6–37,288.8)	27,230.6 (24,699.0–30,612.3)	23,162.8 (20,350.0–26,525.5)	−31.1 (−34.5 to **−**27.4)
Pomorskie	37,296.5 (34,615.8–40,934.5)	26,423.1 (23,638.7–29,753.0)	23,476.0 (20,875.0–26,682.5)	−37.1 (−40.5 to **−**33.5)
Śląskie	38,302.8 (35,354.8–41,841.3)	28,869.1 (26,099.3–32,248.2)	24,839.4 (22,206.8–28,159.0)	−35.1 (−38.3 to **−**32.0)
Świętokrzyskie	31,882.6 (29,194.1–35,399.6)	27,411.3 (24,785.3–30,614.0)	23,934.9 (21,110.3–27,359.4)	−24.9 (−28.8 to **−**21.2)
Warmińsko-Mazurskie	37,193.8 (34,345.5–40,780.6)	28,102.3 (25,524.4–31,456.8)	24,110.5 (21,498.0–27,512.4)	−35.2 (−38.6 to **−**31.5)
Wielkopolskie	36,227.0 (33,333.3–39,771.8)	26,536.4 (23,889.7–29,805.4)	23,418.4 (20,661.3–26,745.9)	−35.4 (−39.0 to **−**31.7)
Zachodniopomorskie	36,273.1 (33,503.8–39,669.4)	27,614.4 (25,027.8–30,830.6)	24,177.2 (21,371.4–27,322.3)	−33.3 (−36.9 to **−**29.8)

DALY, disability-adjusted life-year.

#### Changes in mortality rates in Poland between 1990 and 2023

**Fig. 2:**
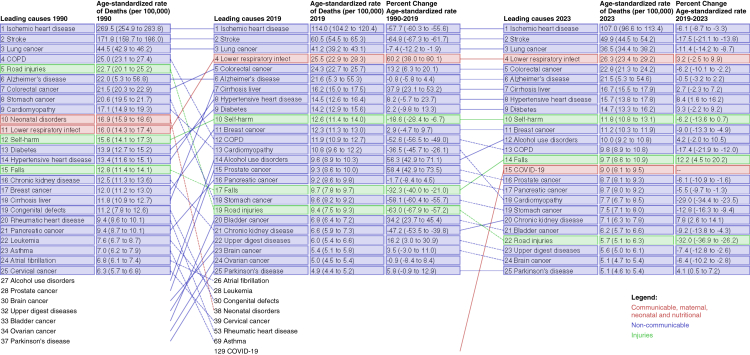
**Relative rank of the leading 20 causes of deaths in 1990 and 2023, change in the number of deaths, and change in the age-standardised rate of deaths between 1990 and 2023.** Data are ranked by Global Burden of Disease Level 3 causes. Causes are connected by arrows between time periods; solid lines are increases in rank and dashed lines are decreases in rank. COPD = chronic obstructive pulmonary disease.

The main causes of death in the analysed period (with exception of COVID-19 in 2020, 2021, and 2022) remained ischaemic heart disease (IHD), stroke, and lung cancers, despite large (18–71%) decreases in age-standardised rates. IHD was the leading cause of death in Poland in 1990 (269.5 age-standardised deaths per 100,000 [95% UI 254.9–283.8]) and in 2023 (107.0 [96.6–113.4]), but in 2021, COVID-19 became the leading cause (168.7 per 100,000 [158.6–173.8]). Between 1990 and 2023, age-standardised mortality rate reductions were observed in other leading causes, as well as in road injuries (1990: 22.7 [20.1–25.2] and 2023: 5.7 [5.1–6.3] deaths per 100,000), and COPD (1990: 25.0 [23.1–27.4] versus 2023: 9.8 [8.9–10.8]) (Fig. 2).

#### Changes in the burden of disease, 2000–2023

There was a notable decline in age-standardised DALYs rates for most causes across Poland and its regions from 2000 to 2023. The largest declines in age-standardised DALY rates among the 20 leading causes are for road injuries (63.2% [95% UI 59.3–66.7]), stroke 56.6 [53.1–59.9]), neonatal disorders (50.4% [44.9–57.4]), IHD (49.4 [46.8–52.5]), congenital birth defects (40.4% [27.6–49.9]), lung cancers (35.3% [31.6–38.6]), and self-harm (25.9% [15.5–33.6]). Nationwide, IHD DALY rates declined from 3761.6 DALYs (3571.1–3944.3) per 100,000 in 2000 to 1903.2 DALYs (1768.1–2002.2) per 100,000 in 2023, and all regions showed substantial declines, with the largest decrease in Mazowieckie, by 58.5% (55.7–61.5). At the national level, stroke burden decreased from 2375.8 DALYs (2191.9–2526.1) per 100,000 population in 2000 to 1031.1 (941.3–1112.6) per 100,000 population in 2023; the most substantial regional decrease was in Podlaskie, at 67.5 (64.0–70.4). For congenital birth defects, national rates improved from 750.7 DALYs (666.0–826.9) per 100,000 population in 2000 to 446.3 DALYs (387.9–518.2) per 100,000 in 2023, with the largest local decrease in Łódzkie: 44.3% (32.0–54.9). For neonatal disorders, age-adjusted DALYs per 100,000 declined from 889.8 (832.0–956.1) in 2000 to 440.9 (385.2–501.3) in 2023, with the largest local decrease in Wielkopolskie: 55.2% (47.2–62.9), and for road injuries, from 1023.8 DALYs (942.1–1102.1) in 2000 to 376.6 (340.3–417.9) in 2023, with the largest local decrease in Małopolskie: 69.2% (64.9–72.9) (Table 3).

**Table 3:** Top 20 causes of age-standardized DALYs 2023 in Poland and region largest and smallest changes from 2000 to 2023.

Cause	Location	2000	2010	2019	2021	2023	Percent Change 2000–2023
Ischemic heart disease	**Poland**	**3761.6 (3571.1–3944.3)**	**2660.4 (2516.2–2797.7)**	**2096.9 (1960.0–2215.0)**	**1941.6 (1816.7–2041.6)**	**1903.2 (1768.1–2002.2)**	**−49.4 (−52.5 to −46.8)**
	Lubuskie	3483.0 (3237.9–3684.9)	2795.6 (2628.9–2984.9)	2222.5 (2047.8–2371.1)	2763.1 (2595.6–2914.0)	2695.8 (2473.7–2925.3)	−22.5 (−29.5 to −14.5)
	Mazowieckie	3907.8 (3697.1–4137.0)	2497.8 (2340.4–2627.5)	1784.5 (1652.1–1902.4)	1643.9 (1532.0–1742.1)	1620.6 (1490.8–1728.2)	−58.5 (−61.5 to −55.7)
Low back pain	Poland	1450.6 (1023.0–1955.6)	1423.1 (1004.2–1921.5)	1424.3 (1002.3–1908.7)	1420.3 (1002.9–1900.1)	1408.2 (996.1–1890.9)	−2.9 (−4.1 to −1.7)
	Zachodniopomorskie	1463.1 (1034.5–1976.0)	1446.0 (1028.6–1949.5)	1451.7 (1017.8–1956.1)	1447.0 (1014.9–1961.0)	1434.1 (1012.2–1946.5)	−1.9 (−5.7 to 2.1)
	Slaskie	1480.2 (1047.7–2014.3)	1438.3 (1021.2–1915.9)	1434.2 (1006.0–1929.0)	1429.9 (1006.7–1929.1)	1415.8 (994.9–1902.5)	−4.3 (−7.7 to 0.2)
Stroke	Poland	2375.8 (2191.9–2526.1)	1592.0 (1480.2–1694.3)	1184.0 (1096.6–1266.5)	1128.2 (1037.5–1206.1)	1031.1 (941.3–1112.6)	−56.6 (−59.9 to −53.1)
	Lubuskie	2470.0 (2273.1–2654.6)	1674.8 (1529.7–1798.8)	1289.7 (1177.1–1390.3)	1337.2 (1223.5–1434.9)	1215.8 (1086.8–1322.9)	−50.7 (−54.8 to −46.0)
	Podlaskie	2696.0 (2490.9–2879.7)	1967.9 (1827.2–2110.8)	1233.9 (1133.9–1325.3)	915.1 (830.8–985.5)	876.6 (782.8–962.3)	−67.5 (−70.4 to −64.0)
Diabetes mellitus	**Poland**	**854.5 (669.5–1076.5)**	**772.4 (605.5–957.0)**	**880.2 (694.2–1083.5)**	**905.6 (714.5–1114.2)**	**924.0 (726.4–1134.1)**	**8.4 (1.1–15.8)**
	Lodzkie	657.3 (493.3–864.4)	738.0 (560.9–939.1)	864.2 (660.9–1087.3)	882.9 (672.9–1118.3)	911.2 (696.1–1136.4)	39.0 (29.1–49.0)
	Zachodniopomorskie	1081.0 (835.2–1398.5)	795.6 (623.2–985.8)	850.2 (655.1–1057.6)	882.2 (685.6–1089.2)	885.0 (678.5–1094.2)	−17.9 (−24.0 to −11.5)
Falls	**Poland**	**885.9 (716.3–1106.7)**	**855.8 (687.4–1085.4)**	**816.4 (645.9–1028.8)**	**856.3 (683.7–1072.9)**	**878.0 (700.7–1106.3)**	**−0.9 (−6.3 to 4.2)**
	Slaskie	730.0 (575.3–922.6)	787.4 (622.4–1019.8)	736.1 (570.5–945.5)	821.7 (648.5–1049.0)	866.2 (684.6–1103.7)	18.7 (11.3–26.3)
	Warminsko-Mazurskie	891.4 (713.8–1122.4)	869.1 (696.7–1109.1)	767.6 (609.8–968.9)	766.1 (606.2–969.9)	774.1 (612.0–985.6)	−13.2 (−18.4 to −9.2)
Tracheal, bronchus, and lung cancer	**Poland**	**1294.8 (1238.8–1347.9)**	**1175.6 (1133.7–1219.3)**	**970.0 (929.6–1021.2)**	**863.9 (824.3–906.2)**	**837.0 (794.1–881.1)**	**−35.3 (−38.6 to −31.6)**
	Zachodniopomorskie	1177.7 (1079.8–1282.1)	1137.2 (1060.6–1225.1)	994.6 (928.2–1061.4)	893.6 (846.2–968.4)	863.6 (789.3–946.5)	−26.5 (−35.3 to −17.2)
	Podkarpackie	1478.5 (1374.5–1590.7)	1293.1 (1213.2–1364.7)	912.6 (862.7–969.2)	852.2 (807.8–897.7)	831.0 (762.8–901.0)	−43.7 (−49.3 to −37.3)
Alcohol use disorders	**Poland**	**532.3 (450.6–644.0)**	**579.7 (495.0–690.7)**	**661.7 (568.3–782.1)**	**678.5 (586.4–805.6)**	**682.2 (587.2–811.4)**	**28.4 (19.5–38.6)**
	Lodzkie	471.4 (387.6–584.1)	674.1 (580.6–777.4)	807.9 (697.2–943.1)	854.5 (743.4–990.5)	847.2 (733.0–986.4)	80.5 (59.0–105.5)
	Opolskie	719.3 (625.0–839.2)	732.3 (634.6–853.9)	823.9 (721.0–958.4)	781.9 (679.8–908.9)	780.7 (664.6–916.5)	8.7 (−4.1 to 22.6)
Cirrhosis and other chronic liver diseases	**Poland**	**504.9 (466.9–551.9)**	**546.8 (508.4–587.8)**	**587.6 (541.9–634.0)**	**629.8 (583.4–676.3)**	**607.6 (565.1–653.9)**	**20.5 (5.9–33.1)**
	Slaskie	409.3 (363.3–460.6)	513.0 (462.6–568.0)	516.8 (467.4–568.1)	599.5 (540.6–654.4)	631.9 (560.7–709.4)	55.0 (30.4–81.1)
	Podkarpackie	520.2 (477.1–572.5)	526.8 (478.8–573.4)	553.2 (507.0–605.9)	568.0 (522.2–617.1)	524.0 (475.4–569.2)	0.9 (−10.8 to 12.5)
Age-related and other hearing loss	**Poland**	**593.0 (421.7–797.8)**	**598.6 (424.8–808.4)**	**595.0 (420.2–800.9)**	**592.9 (418.5–797.5)**	**593.9 (421.4–798.5)**	**0.1 (−0.9 to 1.3)**
	Lubuskie	590.9 (419.6–787.3)	598.4 (422.2–806.4)	594.9 (421.0–803.9)	592.6 (420.3–797.4)	594.3 (419.4–803.3)	0.6 (−2.0 to 3.1)
	Lodzkie	593.6 (423.5–795.6)	597.8 (424.0–803.6)	593.7 (420.7–802.0)	592.0 (420.7–797.2)	591.5 (421.2–788.3)	−0.4 (−2.9 to 2.0)
Lower respiratory infections	**Poland**	**451.2 (407.8–494.6)**	**396.9 (362.9–433.6)**	**562.0 (510.8–617.1)**	**556.2 (501.6–613.2)**	**584.4 (525.3–647.2)**	**29.9 (12.4–50.9)**
	Lodzkie	352.7 (316.0–390.5)	325.8 (296.8–357.3)	529.8 (481.0–588.2)	555.0 (500.6–612.8)	636.7 (562.4–717.2)	81.1 (53.5–112.5)
	Podkarpackie	503.8 (448.1–556.0)	400.8 (366.7–436.9)	595.9 (536.0–656.5)	565.1 (509.7–623.6)	560.8 (499.4–623.8)	11.7 (−6.9 to 31.6)
Self-harm	**Poland**	**780.4 (731.5–854.7)**	**740.6 (664.0–806.5)**	**605.7 (550.0–668.0)**	**608.7 (552.5–671.3)**	**577.5 (526.2–637.4)**	**−25.9 (−33.6 to −15.5)**
	Warminsko-Mazurskie	715.3 (650.5–808.2)	725.5 (637.9–806.9)	653.5 (587.5–728.9)	633.4 (563.4–700.2)	612.0 (533.4–697.0)	−14.2 (−28.5 to 0.2)
	Malopolskie	880.7 (814.0–962.8)	809.0 (724.8–886.4)	610.3 (550.0–672.2)	525.5 (471.3–583.6)	521.2 (464.2–595.1)	−40.7 (−49.0 to −30.5)
Headache disorders	**Poland**	**563.3 (393.5–774.9)**	**563.6 (395.1–779.1)**	**563.2 (394.5–775.2)**	**560.5 (393.1–773.7)**	**560.9 (394.4–775.5)**	**−0.4 (−1.4 to 0.5)**
	Mazowieckie	564.6 (395.3–772.4)	564.6 (397.9–778.4)	564.9 (390.7–778.2)	562.4 (392.1–766.6)	563.9 (396.8–778.8)	−0.1 (−2.7 to 2.5)
	Podkarpackie	562.0 (396.3–769.2)	561.3 (393.8–777.9)	561.2 (392.1–771.5)	558.0 (389.0–769.8)	557.8 (392.6–769.0)	−0.7 (−3.3 to 2.0)
Anxiety disorders	**Poland**	**399.8 (249.1–589.7)**	**411.7 (266.5–601.0)**	**387.1 (237.9–569.9)**	**456.6 (281.7–671.3)**	**527.3 (326.7–785.4)**	**33.1 (−1.0 to 79.8)**
	Lubelskie	399.5 (251.4–594.9)	411.4 (266.0–602.2)	386.7 (238.0–574.7)	459.3 (284.7–670.0)	532.2 (326.8–798.0)	34.5 (0.6–81.9)
	Pomorskie	399.9 (248.3–593.6)	411.5 (266.1–601.8)	387.2 (236.9–573.9)	456.0 (286.8–671.7)	524.6 (324.5–788.4)	32.4 (−1.5 to 80.7)
Colon and rectum cancer	**Poland**	**565.3 (531.9–610.3)**	**568.9 (539.1–595.9)**	**524.0 (494.8–550.7)**	**495.0 (471.2–518.4)**	**494.4 (466.6–521.6)**	**−12.4 (−19.3 to −4.6)**
	Kujawsko-Pomorskie	475.0 (437.3–521.8)	544.8 (505.4–596.6)	496.3 (461.4–541.6)	506.5 (469.2–542.5)	518.0 (467.5–568.8)	9.2 (−4.5 to 22.4)
	Podkarpackie	599.7 (554.2–659.7)	589.8 (556.3–622.9)	445.6 (413.8–474.9)	427.7 (395.5–456.0)	431.5 (388.1–470.5)	−27.9 (−36.6 to −18.3)
Congenital birth defects	**Poland**	**750.7 (666.0–826.9)**	**536.6 (477.6–628.8)**	**446.9 (389.6–524.4)**	**465.9 (407.9–545.2)**	**446.3 (387.9–518.2)**	**−40.4 (−49.9 to −27.6)**
	Lubelskie	796.4 (700.6–890.5)	594.3 (535.9–671.3)	507.6 (444.8–579.9)	535.4 (469.7–606.0)	512.1 (445.7–584.9)	−35.5 (−45.1 to −23.1)
	Łódzkie	738.6 (654.8–831.1)	511.4 (442.7–610.2)	420.7 (358.2–500.9)	432.5 (370.9–518.2)	410.0 (342.8–489.7)	−44.3 (−54.9 to −32.0)
Depressive disorders	**Poland**	**366.5 (250.9–495.6)**	**360.9 (252.3–492.5)**	**361.2 (251.6–501.9)**	**448.6 (314.6–631.4)**	**442.7 (308.2–621.7)**	**21.1 (4.3–42.4)**
	Śląskie	670.7 (445.8–910.0)	698.3 (478.5–974.2)	696.2 (477.5–996.7)	879.6 (598.3–1265.6)	865.4 (585.9–1248.8)	29.5 (6.9–57.3)
	Podkarpackie	329.0 (226.6–448.1)	318.0 (224.0–435.1)	318.1 (219.7–440.1)	394.2 (276.2–546.5)	388.6 (270.2–538.5)	18.4 (2.3–38.2)
Neonatal disorders	**Poland**	**889.8 (832.0–956.1)**	**595.2 (537.8–656.1)**	**454.3 (393.6–511.7)**	**459.1 (399.9–515.2)**	**440.9 (385.2–501.3)**	**−50.4 (−57.4 to −44.9)**
	Kujawsko-Pomorskie	848.5 (756.8–936.3)	600.3 (521.9–677.9)	503.5 (429.4–581.7)	512.9 (438.9–601.5)	492.9 (418.1–582.1)	−41.8 (−52.4 to −30.1)
	Wielkopolskie	959.6 (858.1–1052.8)	663.3 (583.7–743.7)	458.0 (391.8–529.2)	444.1 (377.2–507.1)	429.1 (365.1–502.5)	−55.2 (−62.9 to −47.2)
Alzheimer's disease and other dementias	**Poland**	**387.9 (172.7–833.3)**	**384.3 (172.3–803.0)**	**384.8 (170.1–818.1)**	**377.4 (166.1–809.0)**	**380.4 (169.1–805.4)**	**−1.7 (−4.5 to 1.1)**
	Świętokrzyskie	366.8 (167.7–756.4)	382.0 (173.7–808.6)	383.9 (171.4–793.3)	382.6 (170.8–813.1)	388.9 (168.3–800.7)	6.0 (−0.2 to 13.1)
	Podlaskie	394.2 (170.0–845.7)	386.6 (177.2–817.4)	385.4 (172.0–799.2)	375.3 (167.8–807.3)	367.6 (167.1–758.6)	−6.1 (−12.6 to −0.3)
Road injuries	**Poland**	**1023.8 (942.1–1102.1)**	**671.7 (615.9–736.6)**	**506.9 (462.7–554.0)**	**425.6 (385.8–467.7)**	**376.6 (340.3–417.9)**	**−63.2 (−66.7 to −59.3)**
	Wielkopolskie	1068.2 (988.4–1162.7)	681.0 (621.5–748.9)	556.8 (505.5–609.1)	500.9 (451.9–552.2)	470.4 (415.2–526.5)	−55.9 (−60.5 to −50.5)
	Malopolskie	817.0 (738.7–893.4)	505.8 (453.3–561.7)	337.9 (305.3–372.4)	276.6 (247.7–309.2)	251.4 (220.8–282.6)	−69.2 (−72.9 to −64.9)
COVID-19	**Poland**	**0.0 (0.0–0.0)**	**0.0 (0.0–0.0)**	**0.1 (0.1–0.1)**	**3734.6 (3458.1–4217.5)**	**364.5 (213.9–634.4)**	**– (– to –)**
	Świętokrzyskie	0.0 (0.0–0.0)	0.0 (0.0–0.0)	0.1 (0.0–0.1)	3765.7 (3486.6–4250.9)	354.6 (203.4–621.5)	– (– to –)
	Mazowieckie	0.0 (0.0–0.0)	0.0 (0.0–0.0)	0.1 (0.1–0.1)	3474.7 (3182.3–3975.5)	369.7 (208.0–648.8)	– (– to –)

The bold font indicates the reference value, which is the rate for the whole country.

DALY, disability-adjusted life-year.

Increasing trends in age-standardised DALY rate of at least 8% since 2000 were observed for diabetes, alcohol use disorders, cirrhosis and other chronic liver diseases, lower respiratory infections, and anxiety and depressive disorders. Diabetes was the fourth-leading cause of DALYs in Poland in 2023, with its age-standardised DALY rate increasing by 8.4% (95% UI 1.1–15.8) from 2000; the largest regional increase occurred in Łódzkie, at 39.0% (29.1–49.0)). Alcohol use disorders have also been increasing in Poland: the age-standardised DALY rate was 532.3 per 100,000 (450.6–644.0) in 2000 and 682.2 (587.2–811.4) in 2023. Anxiety and depressive disorders age-standardised DALY rate increased between 2000 and 2023 by 33.1% (−1.0 to 79.8) and 21.1% (4.3–42.4), respectively, reaching 527.3 (326.7–785.4) for anxiety disorders and 442.7 (308.2–621.7) for depressive disorders, though the change in anxiety disorder was not statistically significant. However, the entirety of these increases occurred during and after the COVID-19 pandemic (2019–2023). COVID-19 DALYs in 2021 were nearly equal to DALYs from IHD, stroke, and lung cancer combined (Table 3).

### Age-standardised YLLs in Poland—areas for improvement of life expectancy

**Fig. 3:**
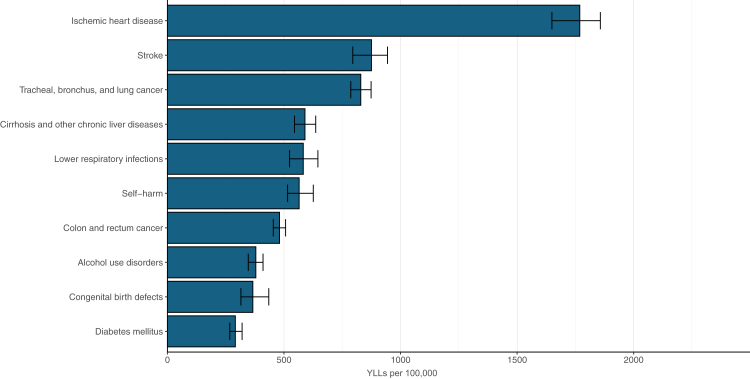
**Top ten age-standardised YLLs rates in Poland in 2023 as areas for improvement.** YLLs = years of life lost.

In 2023, major cardiovascular diseases like IHD and stroke caused 1768.7 (95% UI 1649.4–1857.2) and 875.3 (795.0–943.8) age-standardised YLLs per 100,000 population, respectively. Neoplasms also led to high YLLs (tracheal, bronchus, and lung cancer: 829.4 [786.4–873.5]; cirrhosis and other chronic liver disease: 589.9 [545.1–636.0]) (Fig. 3).

### Risk factors in Poland and its 16 voivodships

Of the leading ten risk factors based on age-standardised risk-attributable DALY rates, high body-mass index and diet high in sodium had non-significant changes between 1990 and 2023 at the national level, at −13.6% (95% UI –32.3 to 6.0) and −55.0 (−83.5 to 44.4). The smallest drop in the age-standardised DALY rate attributable to the top ten risk factors was observed for high fasting plasma glucose, with a 27.0% decrease (14.4–40.9) nationally, and the smallest regional decline, a percentage change of −15.7% (−30.7 to −1.0), in Dolnośląskie (in Świętokrzyskie non-significant decrease of 11.4%). However, the greatest decreases were observed for particulate matter pollution (77.9% [70.9–83.4]) and high LDL cholesterol (70.9% [67.7–74.2]) nationally, and regionally for particulate matter pollution in Zachodniopomorskie (86.1% [79.7–91.5]) and high LDL cholesterol in Łódzkie (77.3% [74.3–80.2]). Greatest increases in age-standardised risk-attributable DALYs are observed for high alcohol use, 35.2% increase (3.5–65.1) at the national level, with the greatest increase seen in Świętokrzyskie, 63.2% (24.8–99.7). Smoking was the leading risk factor in Poland in 2023, resulting in 2269.4 age-standardised DALYs (1853.3–2712.3) per 100,000 and a 58.6% (54.4–63.2) decrease nationally compared to 1990. In 2023, regional disparities in DALYs from risk factors were observed, with Podkarpackie having the lowest DALYs for smoking (1897.3 DALYs [ 1531.5–2294.3] per 100,000), whereas Lubuskie had the highest age-standardised DALYs per 100,000 population due to high blood pressure (2606.8 DALYs [2063.6–3043.8] per 100,000), high LDL cholesterol (1316.5 DALYs [897.7–1759.4] per 100,000), and high fasting plasma glucose (1912.5 DALYs [1628.6–2273.4] per 100,000) (Table 4; Appendix 1, Table S2)

**Table 4:** Leading five risk factors in 1990 and 2023 in Poland by voivodship and European regions and the change in the age-standardised DALYs rate between 1990 and 2023.

Year	Smoking			High systolic blood pressure			High body-mass index			High fasting plasma glucose			High alcohol use		
	1990	2023	% change from 1990	1990	2023	% change from 1990	1990	2023	% change from 1990	1990	2023	% change from 1990	1990	2023	% change from 1990
Western Europe	2988.5 (2489.5–3498.9)	1412.7 (1102.2–1729.0)	−52.7 (−57.5 to −48.0)	2928.0 (2469.8–3353.4)	1071.5 (868.8–1235.4)	−63.4 (−67.2 to −59.6)	1381.9 (560.7–2203.1)	1192.1 (485.5–1832.9)	−13.7 (−29.6 to 4.3)	1323.7 (1113.0–1580.6)	974.2 (815.3–1134.4)	−26.4 (−37.5 to −15.0)	1134.3 (934.1–1441.4)	746.0 (609.7–904.4)	−34.2 (−40.7 to −28.5)
Eastern Europe	3643.3 (3080.0–4276.5)	2686.0 (2222.4–3149.8)	−26.3 (−35.5 to −15.5)	6544.4 (5388.5–7636.1)	4271.5 (3391.8–4962.4)	−34.7 (−45.7 to −20.5)	1942.9 (775.0–3163.9)	2082.5 (886.9–3257.2)	7.2 (−12.5 to 28.4)	1175.1 (865.6–1577.7)	1367.5 (1123.0–1743.7)	16.4 (−11.8 to 51.9)	2105.0 (1759.6–2507.6)	2041.7 (1836.2–2356.7)	−3.0 (−10.4 to 6.9)
Central Europe	4483.1 (3652.7–5317.7)	2366.9 (1921.0–2846.1)	−47.2 (−54.1 to −39.4)	6217.7 (5233.5–7081.5)	2844.3 (2379.2–3199.0)	−54.3 (−57.9 to −49.2)	2040.8 (899.4–3246.1)	1872.0 (882.2–2772.3)	−8.3 (−26.0 to 12.5)	1958.4 (1544.2–2669.6)	1692.0 (1384.5–2089.1)	−13.6 (−36.0 to 10.9)	1394.7 (1130.3–1800.1)	1230.3 (1056.2–1462.9)	−11.8 (−21.0 to −3.0)
Poland	5478.3 (4553.1–6330.3)	2269.4 (1853.3–2712.3)	−58.6 (−63.2 to −54.4)	5723.8 (4759.8–6578.3)	2053.7 (1657.2–2362.4)	−64.1 (−67.9 to −58.9)	2226.4 (966.8–3512.2)	1923.4 (839.0–2907.8)	−13.6 (−32.3 to 6.0)	2443.4 (1998.0–3131.8)	1784.1 (1512.3–2114.8)	−27.0 (−40.9 to −14.4)	1062.4 (825.8–1466.2)	1436.9 (1239.7–1678.4)	35.2 (3.5–65.1)
Dolnośląskie	5980.9 (4991.7–6921.8)	2538.2 (2072.5–3065.6)	−57.6 (−63.0 to −52.4)	5984.8 (4913.8–6956.3)	2246.6 (1754.3–2670.6)	−62.5 (−66.9 to −55.9)	2321.1 (986.8–3815.1)	1994.2 (853.5–3078.8)	−14.1 (−32.6 to 8.3)	1960.2 (1611.5–2450.6)	1652.5 (1407.9–1925.8)	−15.7 (−30.7 to −1.0)	1058.2 (796.9–1496.7)	1411.1 (1185.9–1685.3)	33.4 (4.4–66.0)
Kujawsko-Pomorskie	5978.9 (4951.4–6909.0)	2745.5 (2257.9–3269.2)	−54.1 (−59.8 to −48.9)	5653.3 (4581.8–6531.4)	2069.8 (1656.6–2405.3)	−63.4 (−67.5 to −58.0)	2433.4 (1091.4–3774.1)	2063.4 (907.9–3123.8)	−15.2 (−31.2 to 1.7)	2507.8 (2047.1–3255.6)	1834.6 (1542.0–2194.9)	−26.8 (−41.0 to −12.4)	946.9 (715.4–1367.5)	1341.8 (1124.0–1591.1)	41.7 (6.3–75.7)
Łódzkie	5368.9 (4422.5–6288.7)	2220.2 (1788.6–2719.0)	−58.6 (−64.0 to −53.5)	6015.7 (5035.3–6814.9)	2047.5 (1666.0–2374.0)	−66.0 (−70.8 to −60.8)	2175.4 (921.9–3483.8)	1926.1 (840.4–2987.3)	−11.5 (−33.5 to 12.5)	2617.0 (2199.5–3209.0)	1901.5 (1614.1–2272.5)	−27.3 (−39.8 to −14.9)	1263.7 (989.3–1724.8)	1797.8 (1551.0–2104.4)	42.3 (6.7–74.4)
Lubelskie	5193.4 (4307.2–6030.8)	2298.2 (1886.3–2793.6)	−55.7 (−60.7 to −50.8)	5545.1 (4551.6–6456.1)	2274.1 (1819.1–2651.3)	−59.0 (−63.6 to −52.3)	2068.0 (912.1–3227.6)	1991.2 (871.5–3028.4)	−3.7 (−24.8 to 14.7)	2228.5 (1821.4–2911.9)	1858.2 (1558.0–2185.3)	−16.6 (−34.3 to 0.0)	1014.3 (782.5–1370.4)	1394.6 (1165.6–1670.7)	37.5 (14.9–66.4)
Lubuskie	5330.8 (4381.0–6279.5)	2246.8 (1752.6–2776.9)	−57.9 (−64.3 to −51.4)	5874.2 (4741.4–6864.2)	2606.8 (2063.6–3043.8)	−55.6 (−60.9 to −49.6)	2207.9 (967.0–3481.1)	2070.4 (919.4–3145.7)	−6.2 (−25.4 to 14.2)	2470.6 (1968.6–3206.5)	1912.5 (1628.6–2273.4)	−22.6 (−39.6 to −6.5)	1098.9 (863.4–1526.6)	1263.8 (1047.8–1609.7)	15.0 (−4.8 to 35.6)
Małopolskie	4969.9 (4149.2–5824.6)	2094.8 (1640.5–2554.9)	−57.8 (−63.3 to −52.7)	4995.5 (4060.0–5821.8)	1981.5 (1561.2–2305.9)	−60.3 (−64.7 to −54.3)	2029.1 (912.7–3152.6)	1904.6 (812.4–2880.9)	−6.1 (−26.2 to 13.5)	2179.9 (1801.8–2729.6)	1729.3 (1438.1–2084.8)	−20.7 (−36.4 to −5.8)	841.3 (621.9–1188.4)	1157.2 (971.1–1404.5)	37.6 (4.3–73.2)
Mazowieckie	5809.0 (4780.1–6724.2)	2378.0 (1915.4–2843.9)	−59.1 (−63.7 to −54.5)	6293.8 (5285.2–7159.2)	1835.3 (1479.7–2163.2)	−70.8 (−75.1 to −65.5)	2198.2 (945.6–3500.9)	1839.5 (781.2–2873.7)	−16.3 (−38.3 to 8.1)	2605.8 (2135.7–3327.2)	1773.3 (1498.6–2129.8)	−31.9 (−45.7 to −20.1)	1236.9 (958.2–1663.5)	1451.1 (1252.9–1706.4)	17.3 (−7.8 to 43.0)
Opolskie	5166.2 (4279.3–6063.5)	2058.5 (1612.9–2497.5)	−60.2 (−66.7 to −54.0)	5731.3 (4606.8–6715.9)	2048.6 (1680.1–2392.4)	−64.3 (−68.6 to −58.4)	2236.9 (973.4–3503.7)	1971.5 (888.4–2966.8)	−11.9 (−28.3 to 6.2)	2548.1 (2092.0–3245.4)	1861.7 (1553.0–2212.7)	−26.9 (−41.5 to −13.9)	903.8 (662.0–1326.2)	1250.3 (1048.2–1494.1)	38.3 (2.9–74.2)
Podkarpackie	4719.9 (3972.4–5478.2)	1897.3 (1531.5–2294.3)	−59.8 (−65.3 to −54.8)	5508.6 (4521.6–6325.0)	2072.1 (1744.1–2350.8)	−62.4 (−66.6 to −55.5)	2114.1 (980.7–3272.2)	1979.5 (921.2–2960.7)	−6.4 (−26.4 to 20.7)	2327.0 (1907.3–2957.7)	1747.4 (1453.3–2110.8)	−24.9 (−38.4 to −11.7)	839.0 (624.0–1209.9)	1214.7 (1017.0–1439.2)	44.8 (5.4–79.5)
Podlaskie	4413.7 (3677.8–5169.6)	1974.4 (1569.8–2389.6)	−55.3 (−61.4 to −48.9)	4598.0 (3766.2–5382.3)	1927.4 (1552.0–2229.1)	−58.1 (−63.3 to −51.1)	2179.0 (948.5–3363.7)	1940.2 (876.0–2915.9)	−11.0 (−27.2 to 9.3)	2362.5 (1915.7–2969.2)	1667.9 (1400.4–1999.3)	−29.4 (−41.4 to −16.0)	1171.7 (933.3–1538.9)	1532.0 (1309.0–1797.7)	30.7 (9.1–57.6)
Pomorskie	5585.1 (4659.4–6476.4)	2140.7 (1708.5–2587.8)	−61.7 (−66.3 to −56.9)	5565.2 (4500.3–6479.8)	1872.0 (1497.6–2200.1)	−66.4 (−70.4 to −60.9)	2272.1 (981.4–3592.6)	1891.1 (817.5–2802.8)	−16.8 (−32.8 to −0.3)	2749.4 (2298.2–3411.3)	1870.0 (1610.1–2223.6)	−32.0 (−44.4 to −20.1)	1069.8 (813.9–1489.6)	1501.2 (1281.5–1751.6)	40.3 (8.7–75.2)
Śląskie	5747.2 (4780.6–6640.3)	2141.5 (1696.6–2597.7)	−62.7 (−67.8 to −58.3)	6412.6 (5295.9–7412.9)	2212.0 (1773.0–2547.9)	−65.5 (−69.8 to −59.9)	2437.5 (1084.3–3886.2)	1923.6 (886.4–2889.2)	−21.1 (−39.2 to 3.2)	2674.9 (2166.4–3570.6)	1799.6 (1517.3–2122.9)	−32.7 (−47.1 to −18.2)	1120.5 (839.5–1601.0)	1611.9 (1366.3–1932.4)	43.8 (1.1–82.5)
Świętokrzyskie	4317.8 (3509.0–5092.7)	2081.7 (1678.4–2540.2)	−51.8 (−58.5 to −45.3)	4822.3 (3950.0–5633.9)	2363.2 (1913.8–2749.0)	−51.0 (−58.8 to −39.7)	1855.9 (800.1–2968.7)	1950.4 (854.1–2954.5)	5.1 (−17.7 to 30.3)	1932.3 (1590.0–2438.6)	1712.0 (1485.7–1983.0)	−11.4 (−26.0 to 2.5)	927.6 (720.3–1267.2)	1513.7 (1276.2–1765.9)	63.2 (24.8–99.7)
Warmińsko-Mazurskie	5983.8 (5063.8–6938.4)	2361.6 (1931.4–2824.3)	−60.5 (−65.6 to −55.9)	5517.9 (4506.0–6456.4)	1825.4 (1469.0–2137.2)	−66.9 (−71.3 to −61.7)	1995.9 (858.9–3255.0)	1738.9 (744.0–2666.1)	−12.9 (−32.6 to 5.6)	2543.5 (2073.3–3272.0)	1788.9 (1508.1–2121.9)	−29.7 (−42.8 to −17.6)	1159.5 (909.5–1547.4)	1620.0 (1399.9–1866.6)	39.7 (12.6–70.4)
Wielkopolskie	5771.8 (4791.7–6654.9)	2335.4 (1897.9–2802.1)	−59.5 (−64.1 to −54.0)	5977.9 (4853.1–6996.5)	1893.4 (1514.7–2206.8)	−68.3 (−71.9 to −63.3)	2505.5 (1078.1–3945.1)	1883.5 (814.9–2867.9)	−24.8 (−40.7 to −5.7)	2777.2 (2279.0–3604.7)	1790.6 (1510.7–2108.5)	−35.5 (−48.7 to −23.9)	1026.4 (775.5–1484.3)	1342.0 (1139.9–1598.1)	30.8 (−4.8 to 63.7)
Zachodniopomorskie	5976.1 (4997.2–6886.5)	2529.7 (2044.1–3019.1)	−57.7 (−62.5 to −52.8)	5538.6 (4490.7–6453.9)	2069.1 (1646.3–2411.4)	−62.6 (−67.4 to −57.7)	2308.7 (988.8–3666.0)	1910.6 (829.3–2897.1)	−17.2 (−33.7 to 4.5)	2387.8 (1887.5–3182.2)	1737.5 (1476.4–2043.4)	−27.2 (−41.5 to −11.0)	1081.5 (846.5–1471.4)	1547.7 (1339.4–1799.6)	43.1 (8.8–75.0)

DALY, disability-adjusted life-year.

## Discussion

In 2023, Poland's life expectancy was lower than the average for western European countries but higher than that of central and eastern Europe for males and females combined. Life expectancy reached 78.6 years (95% UI 78.5–78.7) in Poland, compared to a global estimate of 73.9 (73.6–74.1). Between 1990 and 2023, life expectancy in Poland increased by 7.4 years, in western Europe 5.8 years, central Europe 6.9 years, and eastern Europe 3.6 years. Regional health disparities in Poland remain a persistent challenge, despite substantial improvements in national health indicators, and are visible in estimates of life expectancy, overall disease burden, and mortality rates. The highest life expectancy was in Małopolskie voivodship, which is similar to Czechia and Croatia, and lowest in Łódzkie, where life expectancy was close to Bosnia and Herzegovina. These patterns reflect broader European trends, where a gap between eastern and western European areas persists both between and within countries driven by historical, socioeconomic, and health policy factors.^5^^,^^6^^,^^13^ The main health problems in Poland were associated with ischaemic heart disease (IHD), stroke, and cancers, all three of which have seen marked declines; however, the scale of improvement varies by region. For instance, the reduction in IHD mortality contributed most to life expectancy gains, but the benefit was greatest in Wielkopolskie and lowest in Podlaskie. Some regions, such as Łódzkie, experienced significant increases in diabetes-related DALYs, illustrating the complexity of regional health trends, which may be partly due to variations in diagnostic criteria and reporting practices but also reflect true differences in disease prevalence and severity.^6^^,^^7^^,^^13^ Cancer mortality also shows regional variation, with higher rates in western Poland and lower rates in the southeast. Disparities in cancer outcomes are influenced by differences in access to and participation in screening, early detection, and quality of care.^8^^,^^13^

Mental health issues represent a growing public health challenge in Poland. The burden of anxiety and depressive disorders has increased, especially during and after the COVID-19 pandemic. Socioeconomic factors, stigma, and uneven access to mental health services contribute to these trends, and mental health inequalities are exacerbated by regional disparities in health-care resources and access, with rural and less affluent regions facing particular challenges.^13^^,^^17^, ^18^, ^19^ Gender differences are also pronounced: women are more frequently diagnosed with depression and anxiety, while men have higher suicide rates, reflecting both social and behavioural factors.^13^^,^^15^ Gender disparities are also a persistent feature of other health outcomes in Poland. Women live longer than men (on average 82 versus 74 years). Men are more likely to die from cardiovascular disease, cancer, and suicide, while women are more likely to experience chronic disability and mental health disorders.^13^^,^^15^ Socioeconomic status and education further amplify these gender gaps. Although women have higher levels of education than men, women in lower socioeconomic groups face greater health risks and barriers to care.^13^^,^^18^^,^^19^ Socioeconomic inequalities, such as differences in educational attainment and income levels, play a key role in NCD risk and outcomes.^18^^,^^19^ These factors, however, do not explain the variation as regions with lower PPS per inhabitant have better health indicators (Appendix 1, Section 1, Figure S12).

In 2023, smoking was a main driver of DALYs for males and females combined, along with the main metabolic risk factors: high blood pressure, high fasting plasma glucose, and high LDL cholesterol. For each of these, a 5–50% drop in risk-attributable age-standardised DALY rates over 1990–2023 was observed. However, age-standardised DALY rates from high alcohol use increased by 35%, in contrast to analysed regions of Europe where they declined. The contribution of particulate matter air pollution to age-standardised DALY rates dropped from third to seventh position, with a decline of about 78% between 1990 and 2023. The regional variability remained: smoking burden has dropped by 58.6% nationally, with the lowest rates in Podkarpackie, but high alcohol use is rising, especially in Świętokrzyskie. These patterns reflect both historical behaviours and the uneven effectiveness of public health policies and suggest the need for strategies better tailored to local health challenges. Since 1990, Poland has undergone extensive political, social, and health-care reforms, many of which have shaped its population's health. In 1999, a major reform introduced health insurance contributions and defined responsibilities for health care across government levels, including hospital ownership. That same year, administrative (assigning health-care duties) and social security reform took place, placing the Social Insurance Institution in charge of managing social benefits and health insurance premium collection. The Ministry of Health (MoH) now serves as the principal regulator and policy maker, while the National Health Fund (NHF) acts as the sole payer, providing universal coverage through social health insurance. Health-care providers either contract with the NHF or offer private services.^42^ Starting in 2022, insurance was widened for Ukrainian refugees, estimated by some to be 1.4–2.0 million displaced people in 2022.^43^ Public health expenses have steadily risen.^44^, ^45^, ^46^ In 2021, a new law gradually increasing public health expenditure for achievement of 7% of GDP in 2027 was introduced.^47^ By 2023, health spending reached 7.05% (using a 2021 GDP baseline) or 5.42% (calculated as a percentage of 2023 GDP).^48^ Absolute public spending on health per capita has increased almost four-fold since 2000 and resulted in 2973 USD PPP in 2022.^49^, ^50^, ^51^ Further increases in spending will be supplemented by the national Recovery and Resilience Plan.^52^, ^53^, ^54^ In 2021, the highest share of health spending was on inpatient care (34%), outpatient care (31%), pharmaceuticals (21%), and long-term care (8%). The share spent on prevention was 2%.^9^ Out-of-pocket payments accounted for 20% of all health costs in 2021.^9^

Between 1990 and 2021, numerous health-care standards and programmes of coordinated care were implemented, including maternal and neonatal, cancer,^55^ cardiac care,^56^ improving integration of primary and secondary care, and considerably better access to innovative therapies, new technology, and infrastructure. Actions taken to improve lifestyle and reduce behavioural risk factors played an important role in diminishing the burden of diseases in some areas of public health in Poland; however, the funding level is disputed. The prevention programmes have low participation rates and are not commonly accessible. Smoking was the leading risk factor in 2023, but attributable burden declined considerably. Lung cancer mortality in males is high but declining. Anti-smoking campaigns and legislation have been particularly successful.^57^, ^58^, ^59^, ^60^ Effective interventions included public education campaigns, advertising bans, sponsorship restrictions, tobacco-free zones, higher tobacco taxes, and innovative screening programmes.^61^^,^^62^ As presented before, alcohol use disorders are high, and the burden attributable to high alcohol use is increasing. Anti-alcohol interventions have had some successes in the past 30 years, including lowering mortality, but there have also been periods of increased burden.^63^, ^64^, ^65^, ^66^, ^67^, ^68^

In the past three decades, Poland has also experienced substantial economic growth, with GDP PPP per capita increasing from 44% of the EU average in 1995 to 80% in 2023.^69^ It has led individuals and families to increase their wealth, which has a strong impact on the reduction in lifestyle-related health risks. There is no single explanation for the variable health outcomes that we find by voivodship.^13^ After 30 years, the differences had changed but remained. The COVID-19 pandemic, which resulted in high loss of life, was the subject of numerous analyses and discussions,^70^^,^^71^ but still there is obvious need for monitoring long-term health consequences of the pandemic.

Contributing factors to Poland's disease burden trends include shifts in health technology access, health-care use, and lifestyle changes.^13^^,^^50^^,^^72^ However, better and more reliable data are needed to fully explain all the health improvements in Poland and central and eastern Europe, as the analysis relied on an ecological approach and used limited sources.^73^, ^74^, ^75^ After several successful decades, Poland now faces challenges associated with stagnating mortality rates and an ageing demographic structure. Major imbalances in the provision of health-care services exist, including high expenditure on hospitalisations and low spending on prevention, a lack of coordination between different levels of care, and prolonged waiting times for patients.^42^ These issues highlight the need for reforms to ensure more efficient and equitable health-care delivery. Based on an analysis of GBD estimates for Poland between 1990 and 2023, a call for action could include a wide strategic approach outlined in Panel 1.

Panel 1 **Strategic Call for Action: Improving health in Poland.**

1. Conduct a comprehensive assessment of regional health needs to address and close the health gap between regions.
2. Implement an operation plan to target major health problems and risk factors, and allocate sufficient funding for public health and prevention, with a focus on improving health literacy across all age groups.
3. Prepare for population ageing by preventing diseases, optimising health-care usage, and redefining patient care pathways based on clinical and organisational effectiveness.
4. Introduce new technologies and rationalise health-care spending through evidence-based medicine and health technology assessments, ensuring that the aim of digitalisation is to enhance patient care and system efficiency.
5. Strengthen data analysis and modelling with multidisciplinary teams to evaluate programmes and policies, addressing challenges like COVID-19, the Russian-Ukrainian war, and cybersecurity to improve health system resilience.^76^, ^77^, ^78^
6. Focusing on health aims,^79^ as well as their indicators as prevention outcomes, diagnosis, treatment effectiveness, and key epidemiological measures like mortality, HALE (healthy life expectancy), and DALYs, while avoiding reliance on the sheer volume of health procedures as a metric for assessing health-care quality.

This study has some important limitations, many of which have been noted in previous GBD 2021 publications.^26^^,^^28^^,^^39^^,^^80^^,^^81^ These include reliance on imperfect cause-of-death data and a high proportion of “garbage codes” (30%) on death certificates.^82^ Significant regional disparities in health-care quality, economic growth, and investment levels further complicate mortality analyses in Poland, leading to variable trends across central and eastern Europe. Limited or poor-quality raw data have caused discrepancies in the model, such as declining fasting plasma glucose trends that contradict rising diabetes prevalence or irregular ischaemic heart disease (IHD) mortality data in Pomorskie, influenced by changes in death-coding personnel. Modelling non-fatal estimates, particularly for COVID-19 and other pandemic outcomes, also proved challenging due to inconsistencies in data quality and completeness. Despite these challenges, the study's primary strength lies in presenting the most comprehensive data on Poland's health indicators, providing consistent estimates across all regions. Despite ongoing digitalisation, Poland's health data still heavily rely on administrative records and lack systematic epidemiological studies and clinical data. GBD 2023 helps address this gap, but more robust health registries and regular epidemiological surveys are still needed. The GBD methodology itself has played a pivotal role in addressing data quality issues. By applying rigorous mathematical recalculations, it has produced more reliable national and regional estimates for 1990 to 2023. For the first time, Poland now has regional-level risk factor estimates spanning the entire analysed period. These GBD-derived estimates have become essential tools in health policy planning. They are included in the MoH's “Maps of Health Needs” database, which integrates epidemiological and health-care data to support national and regional strategies^53^ and was instrumental in developing Poland's strategic national plan^83^, ^84^, ^85^ and aligning with the European Union's priorities and post-COVID-19 challenges plans.^52^^,^^54^^,^^86^

### Conclusions

The GBD Poland subnational analysis provides comprehensive estimates for 375 causes of death and disability across Poland's 16 voivodships from 1990 to 2023. While reductions in cardiovascular diseases, neonatal mortality, and some cancers increased life expectancy, these gains varied by region. The cancer burden decreased less than cardiovascular diseases, with some cancers and conditions like diabetes and alcohol use disorders worsening. The COVID-19 pandemic in 2020–2021 resulted in a transient loss of life expectancy, bringing the total improvement since 1990 to 7.4 years in 2023. GBD estimates are one of the most important tools for analysing health-care needs, shaping health strategy, guiding investments, and informing prevention efforts. This analysis identifies areas for targeted treatment and prevention, helping Poland progress towards western European health standards.

## Contributors

Please see Appendix (Appendix 1, Section 3) for more detailed information about individual author contributions to the research, divided into the following categories: providing data or critical feedback on data sources; developing methods or computational machinery; providing critical feedback on methods or results; drafting the manuscript or revising it critically for important intellectual content; and managing the estimation or publications process. All authors had full access to all the data in the study and had final responsibility for the decision to submit for publication. RPM and MN accessed and verified the data.

## Data sharing statement

This study follows the Guidelines for Accurate and Transparent Health Estimates Reporting (GATHER). To download the data used in these analyses, please visit the Global Health Data Exchange (GHDx) (https://ghdx.healthdata.org/).

## Editor note

The Lancet Group takes a neutral position with respect to territorial claims in published maps and institutional affiliations.

## Declaration of interests

S Bhaskar reports grants or contracts Japan Society for the Promotion of Science (JSPS), Japanese Ministry of Education, Culture, Sports, Science and Technology (MEXT) (Grant-in-Aid for Scientific Research (KAKENHI) Grant ID: 23KF0126), JSPS and the Australian Academy of Science (JSPS International Fellowship Grant ID: P23712); Leadership or fiduciary role in other board, society, committee or advocacy group, paid or unpaid as the visiting director in the department of neurology at the National Cerebral and Cardiovascular Center, Suita (Osaka, Japan), district chair of diversity, equity and inclusion at the Rotary District 9675, chair and manager of the Global Health and Migration Hub Community (Berlin, Germany), an editorial board member of PLOS One, BMC Neurology, Frontiers in Neurology, Frontiers in Stroke, Frontiers in Aging, Frontiers in Public Health & BMC Medical Research Methodology, a member of the College of Reviewers (Canadian Institutes of Health Research, Government of Canada), a member of the scientific review committee at Cardiff University Biobank (UK), an export advisor and reviewer with the Cariplo Foundation (Milan, Italy), a Visiting Director at the National Cerebral and Cardiovascular Center, Department of Neurology, Division of Cerebrovascular Medicine and Neurology (Suita, Osaka, Japan), Director of Research with the World Headache Society (Bengaluru, India), and Healthcare and Medical Adviser of Japan Connect (Osaka, Japan); outside the submitted work. J J Jozwiak reports payment or honoraria for lectures, presentations, speakers bureaus, manuscript writing or educational events at NOVARTIS, ADAMED, AMGEN, BOEHRINGER INGELHEIM, SERVIER, NOVO NORDISC; outside the submitted work. All other authors have declared no interests.

## Appendix A Supplementary data

Supplementary Figures and Tables

Poland Summary
