# Supplementary material for: The burden of diseases, injuries, and risk factors by voivodship in Poland, 1990–2023: a systematic analysis for the Global Burden of Disease Study 2023
Source: Lancet Reg Health Eur. 2025 Sep 3;58:101431. doi: 10.1016/j.lanepe.2025.101431 (PMC12624802; doi:10.1016/j.lanepe.2025.101431)
Supplement: Supplementary Figures and Tables [file mmc1.pdf]

Appendix 1: The burden of diseases, injuries, and risk factors by voivodship in Poland, 1990-2023: a systematic analysis for the Global Burden of Disease Study 2023

This appendix provides additional results for “The burden of diseases, injuries, and risk factors by voivodship in Poland, 1990-2023: a systematic analysis for the Global Burden of Disease Study 2023.”

Table of contents

Section 1: Appendix tables and figures

Figure S1. Change in life expectancy due to ischeamic heart disease deaths in Poland, 1990-2023

Figure S2. Change in life expectancy due to stroke death in Poland, 1990-2023

Figure S3. Change in life expectancy due to neonatal disorders deaths in Poland, 1990-2023

Figure S4. Change in life expectancy due to transport injuries death in Poland, 1990-2023

Figure S5. Change in life expectancy due to unintentional injuries death in Poland, 1990-2023

Figure S6. Change in life expectancy due to chronic respiratory diseases death in Poland, 1990-2023

Figure S7. Change in life expectancy due to self-harm and interpersonal violence death in Poland, 1990-2023

Figure S8. Change in life expectancy due to diabetes and kidney death in Poland, 1990-2023

Figure S9. Change in life expectancy due to unintentional injurie death in Poland, 1990-2023

Figure S10. Gained years of life expectancy in Poland, 1990-~~2023~~

Table 1a. MEDLINE database search via OVID

Table 1b. Embase database search via OVID

Table 1c. Cochrane library search

Figure S11. GBD studies included in Poland results

Figure S12. Regional gross domestic product by NUTS 2 region

Figure S13. Life expectancy with population size by millions in Poland

Table S2. Percentage change in risk-attributable burden by DALYs in Poland between 1990 and 2023

Table S3. Life expectancy decomposition by causes in Poland, 16 voivodships, and European regions between 1990 and 2023.

Section 2: GATHER Compliance: Guidelines for Accurate and Transparent Health Estimates Reporting

Table S4. GATHER table

Section 3: Author's Contributions

Author's Contributions

Figure S1. Change in life expectancy due to Ischemic heart disease death

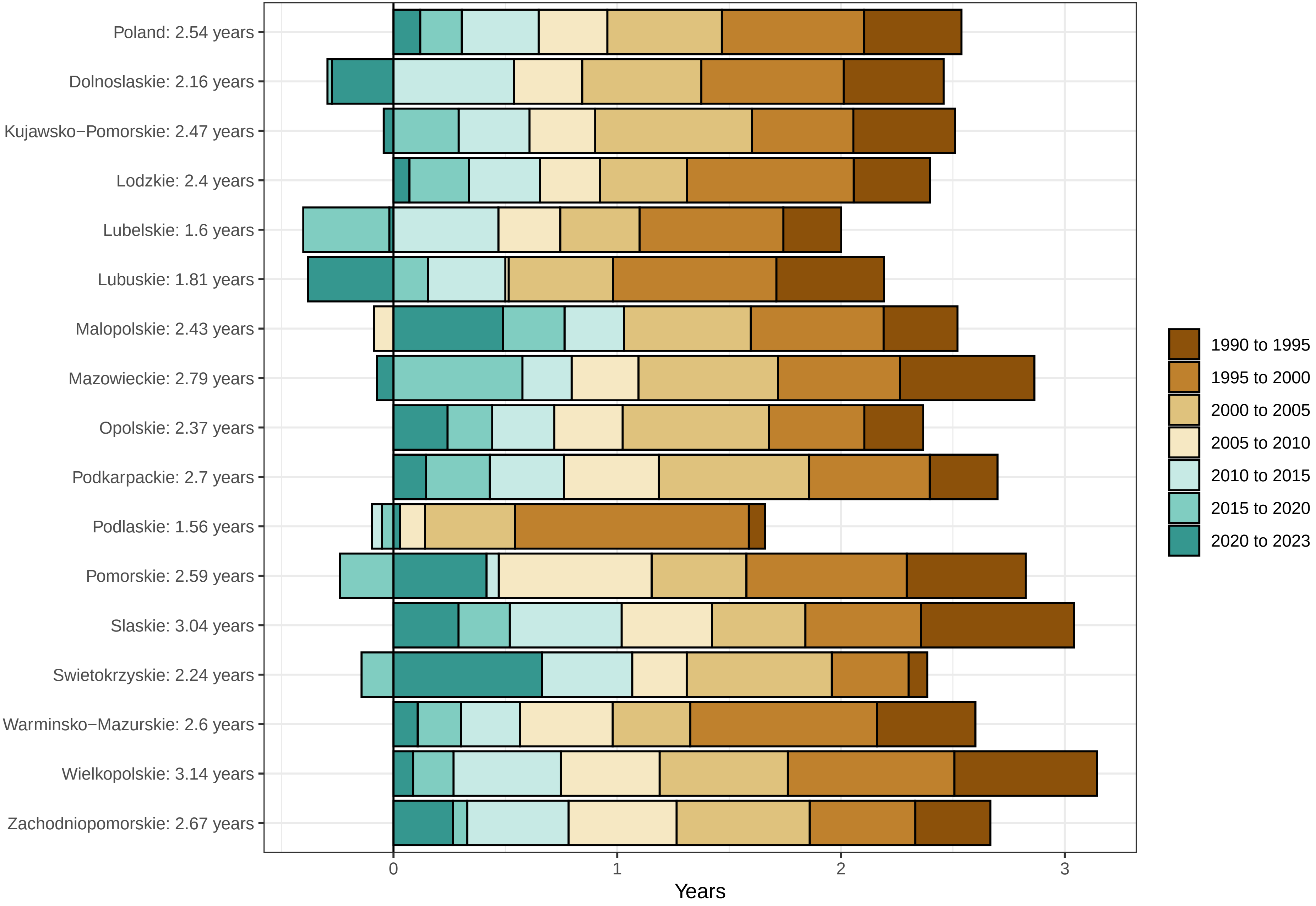

Figure S2. Change in life expectancy due to Stroke death

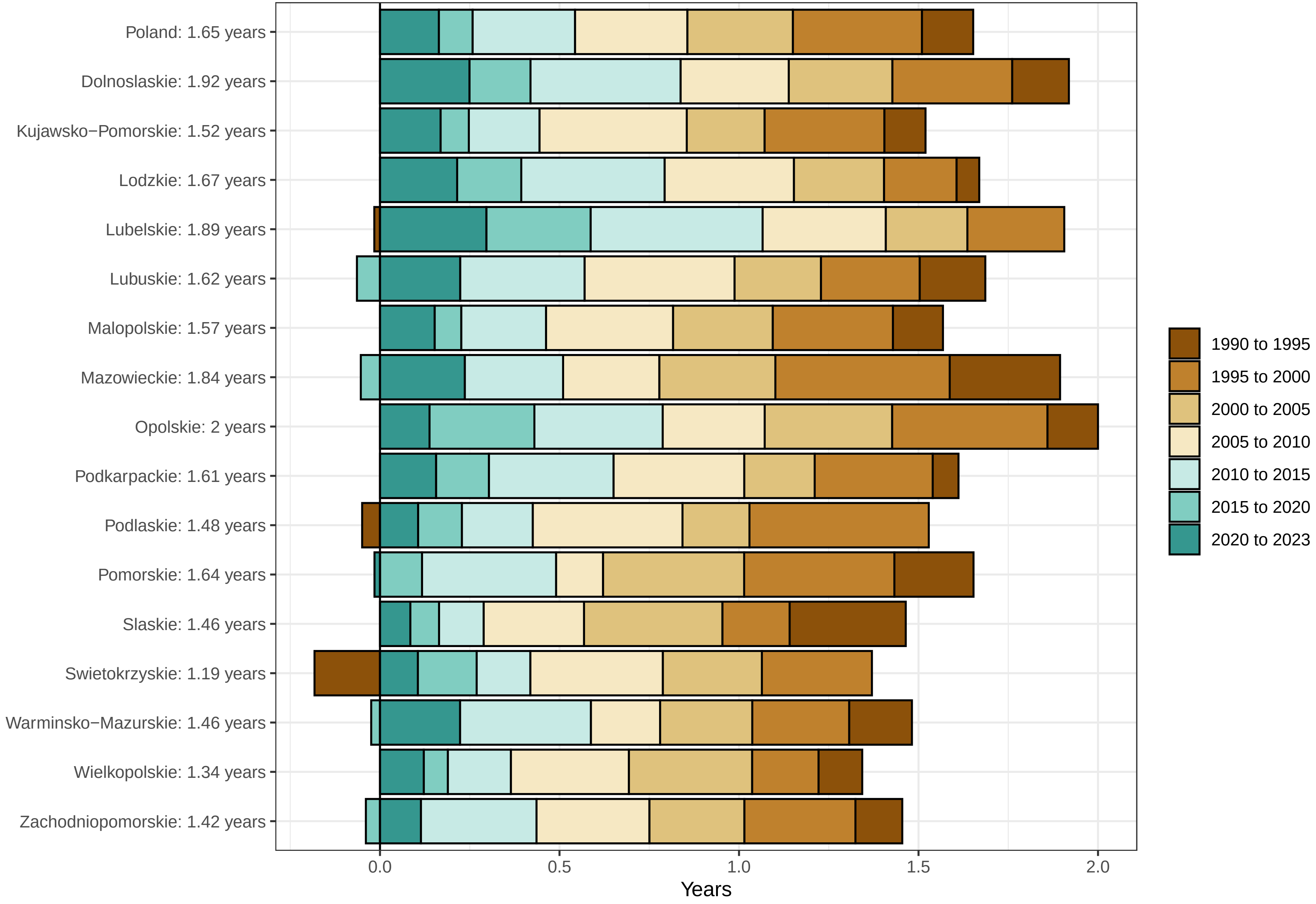

Figure S3. Change in life expectancy due to Neonatal disorders death

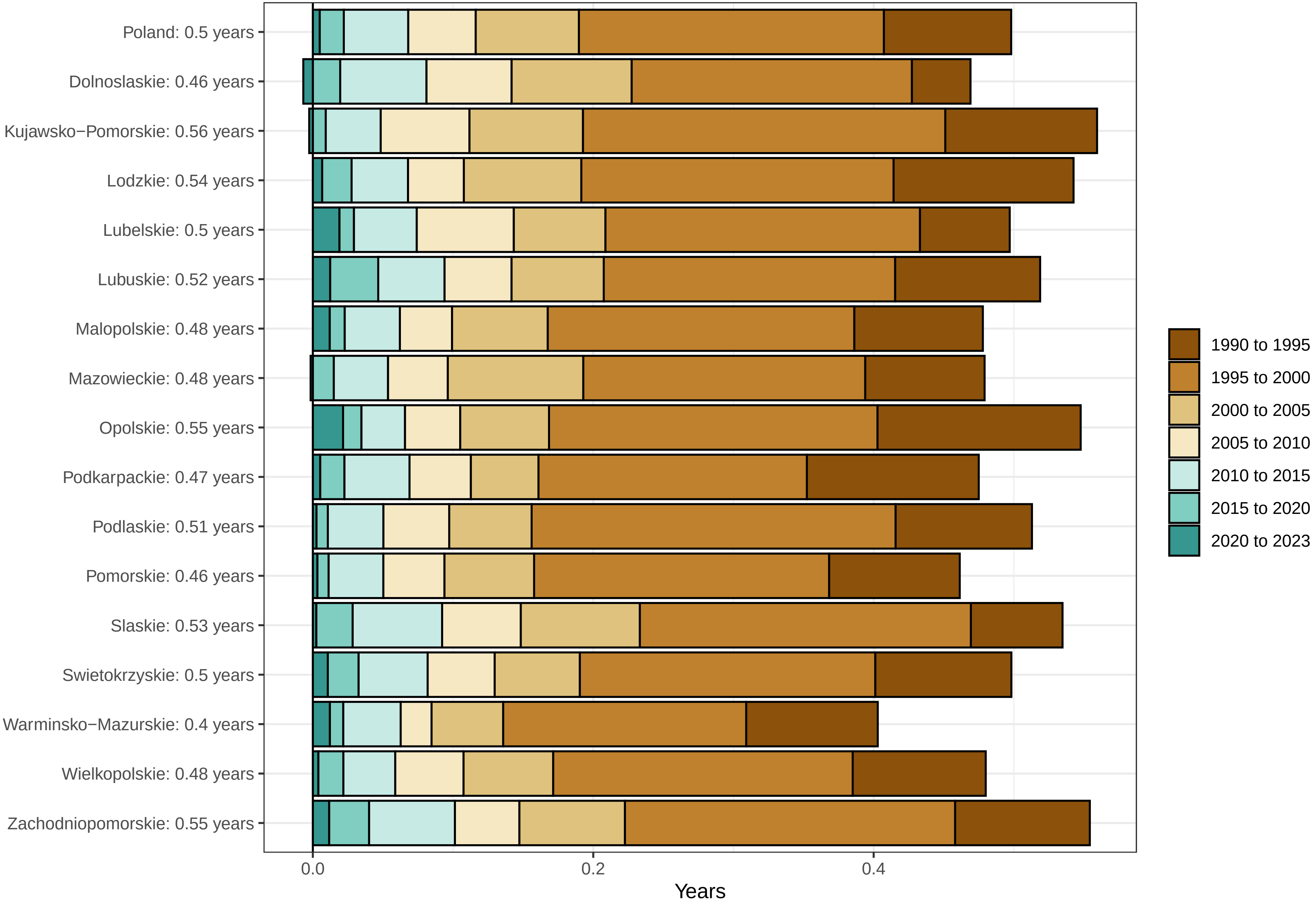

Figure S4. Change in life expectancy due to Transport injuries death

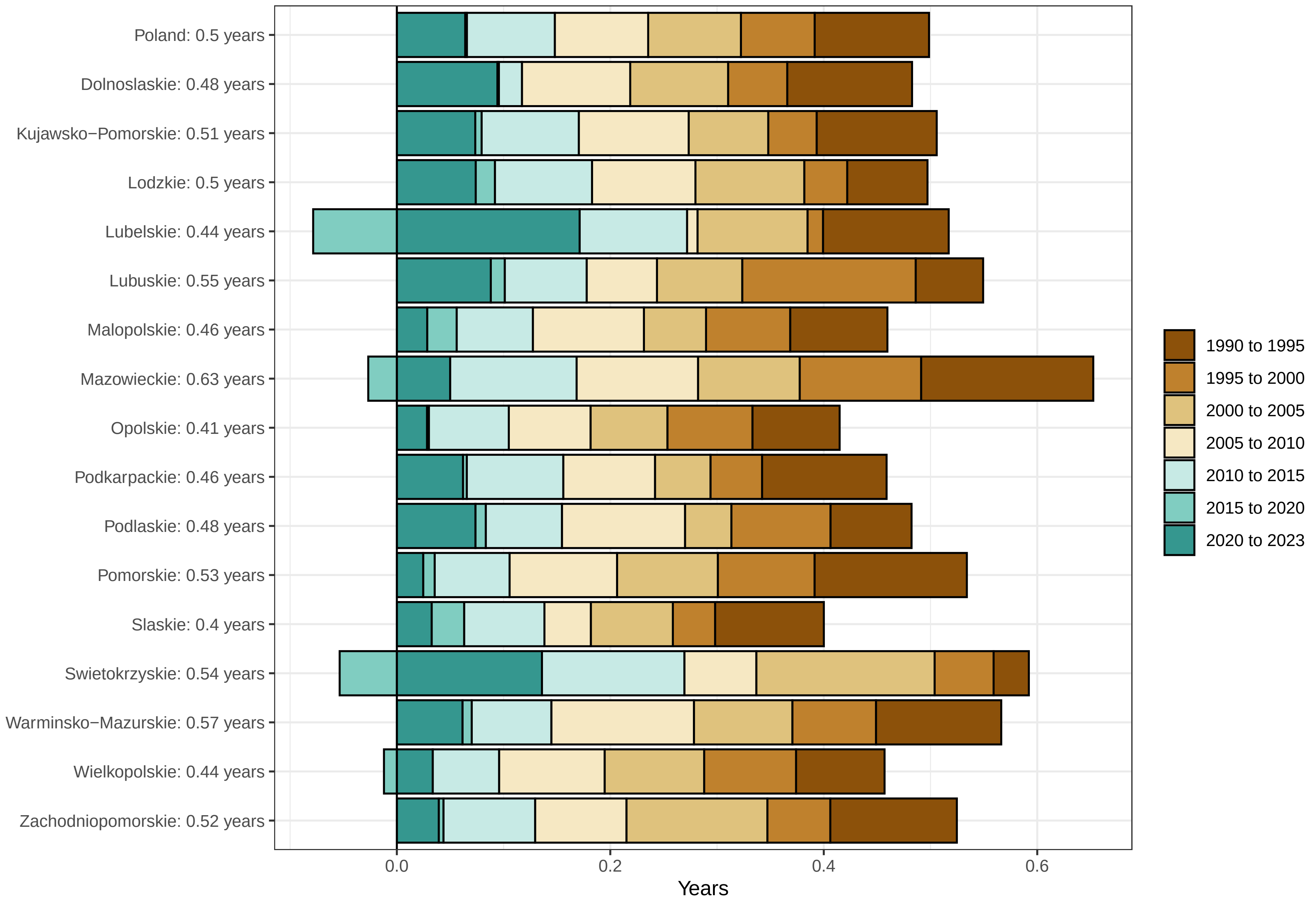

Figure S5. Change in life expectancy due to Unintentional injuries death

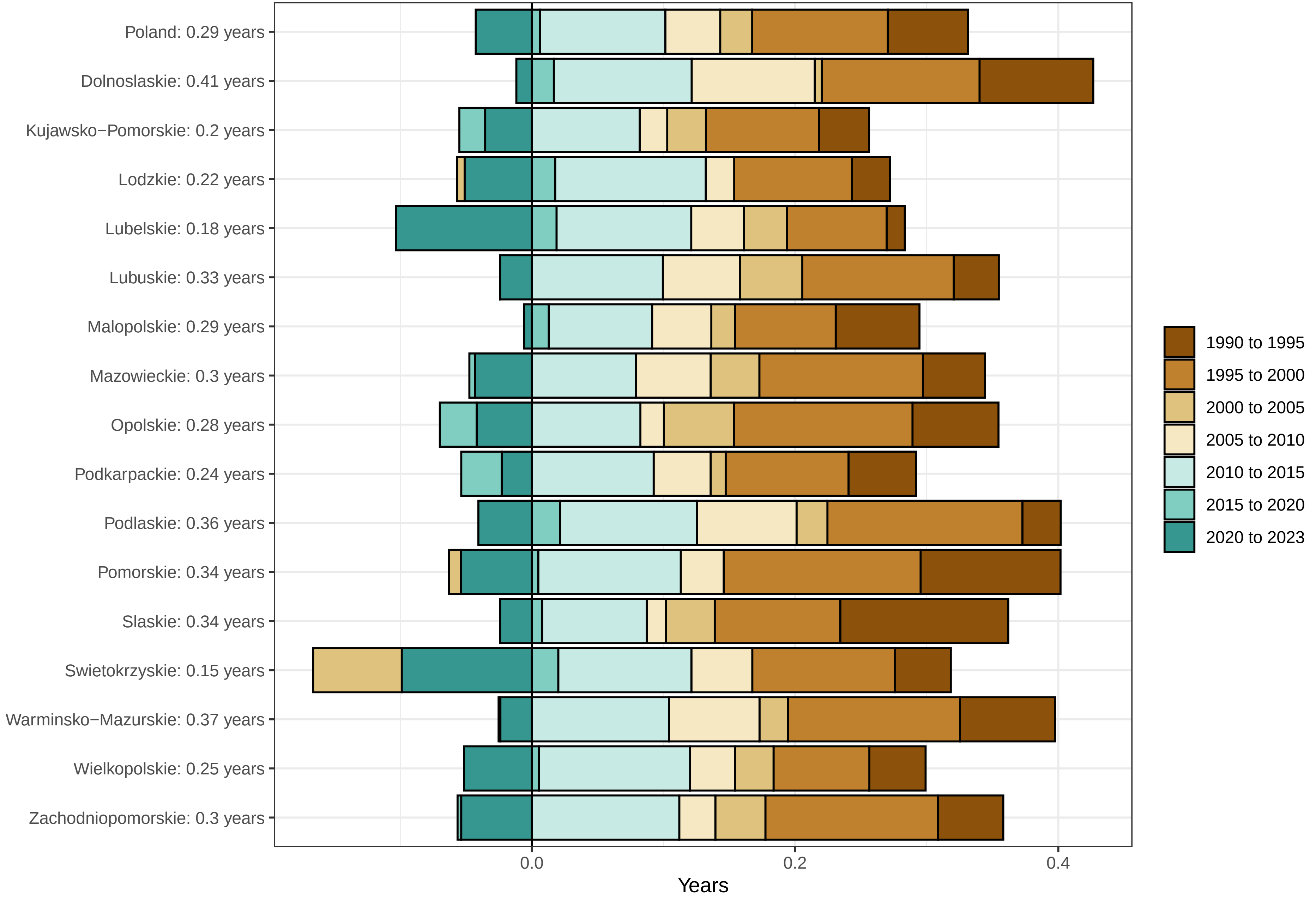

Figure S6. Change in life expectancy due to Chronic respiratory diseases death

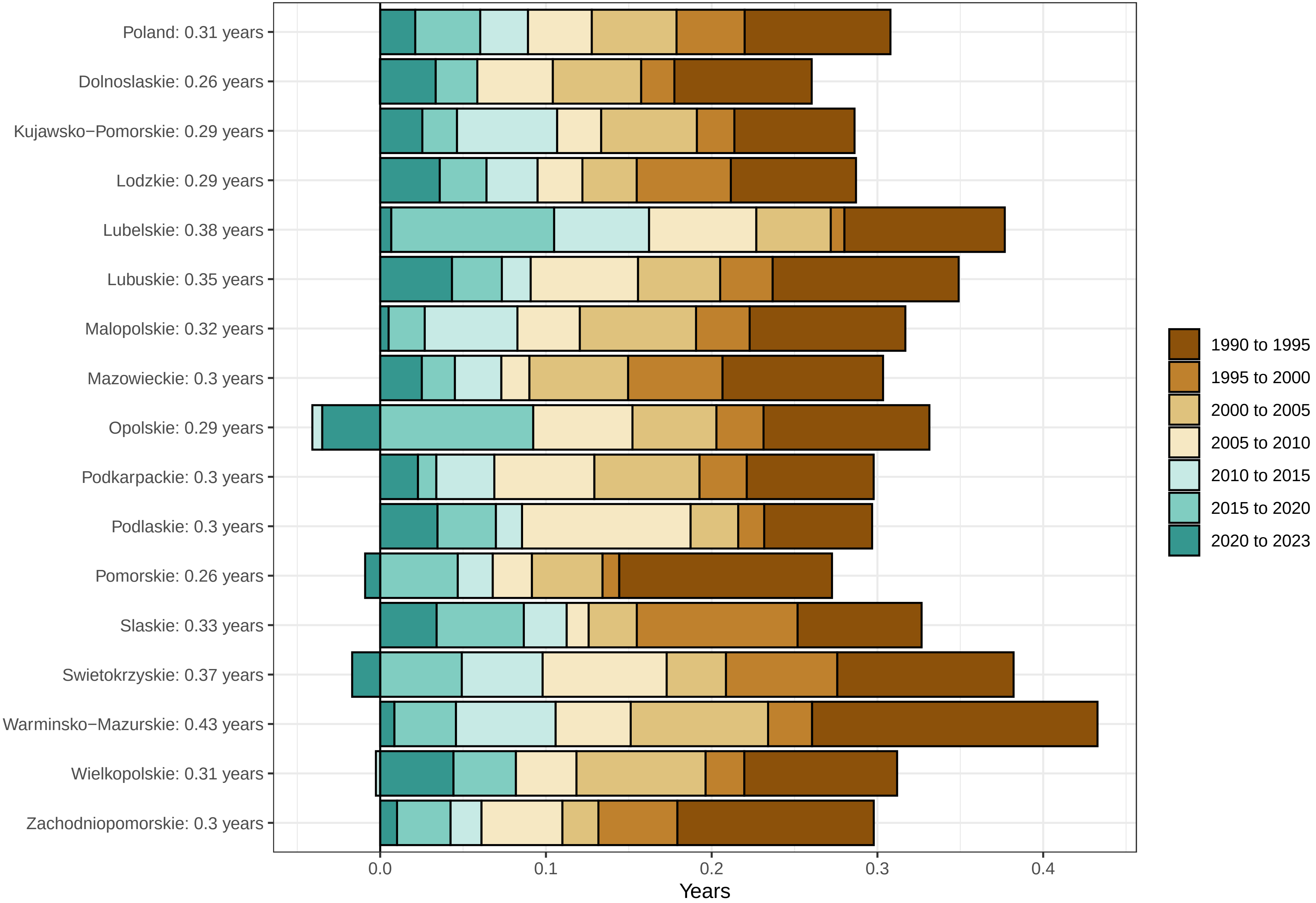

Figure S7. Change in life expectancy due to Self−harm and interpersonal violence death

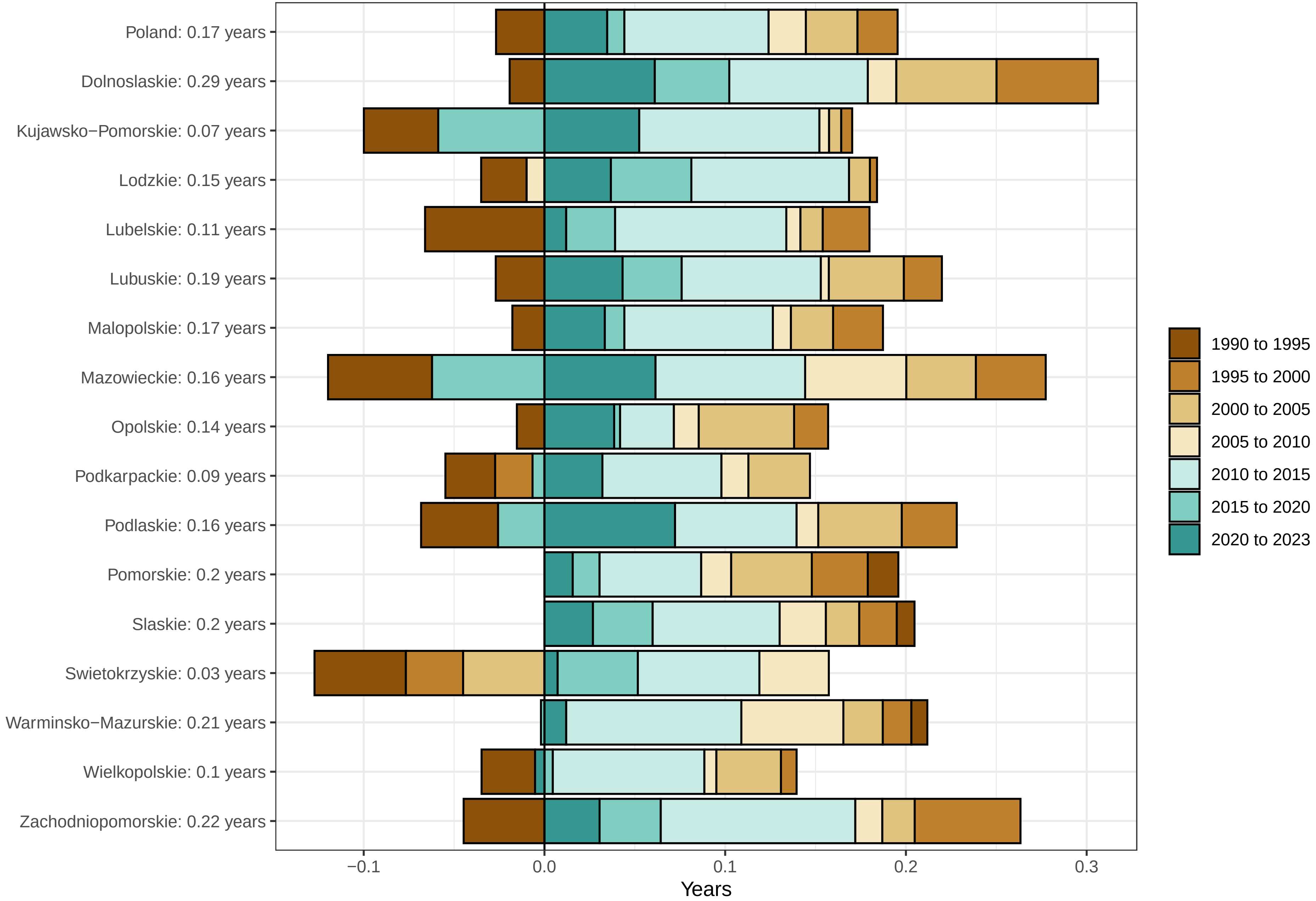

Figure S8. Change in life expectancy due to Diabetes and kidney diseases death

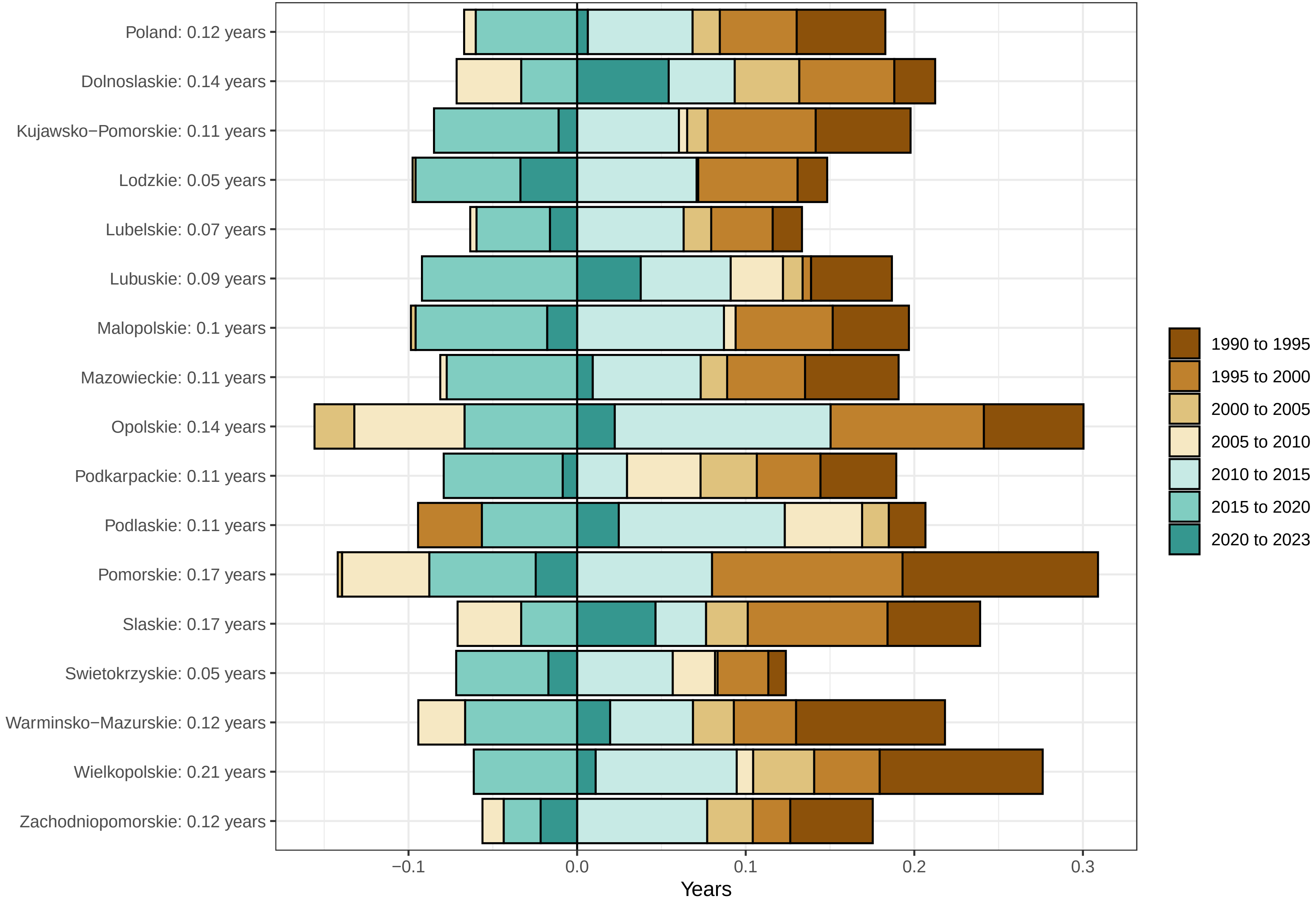

Figure S10. Gained years of life expectancy in Poland, 1990-2021

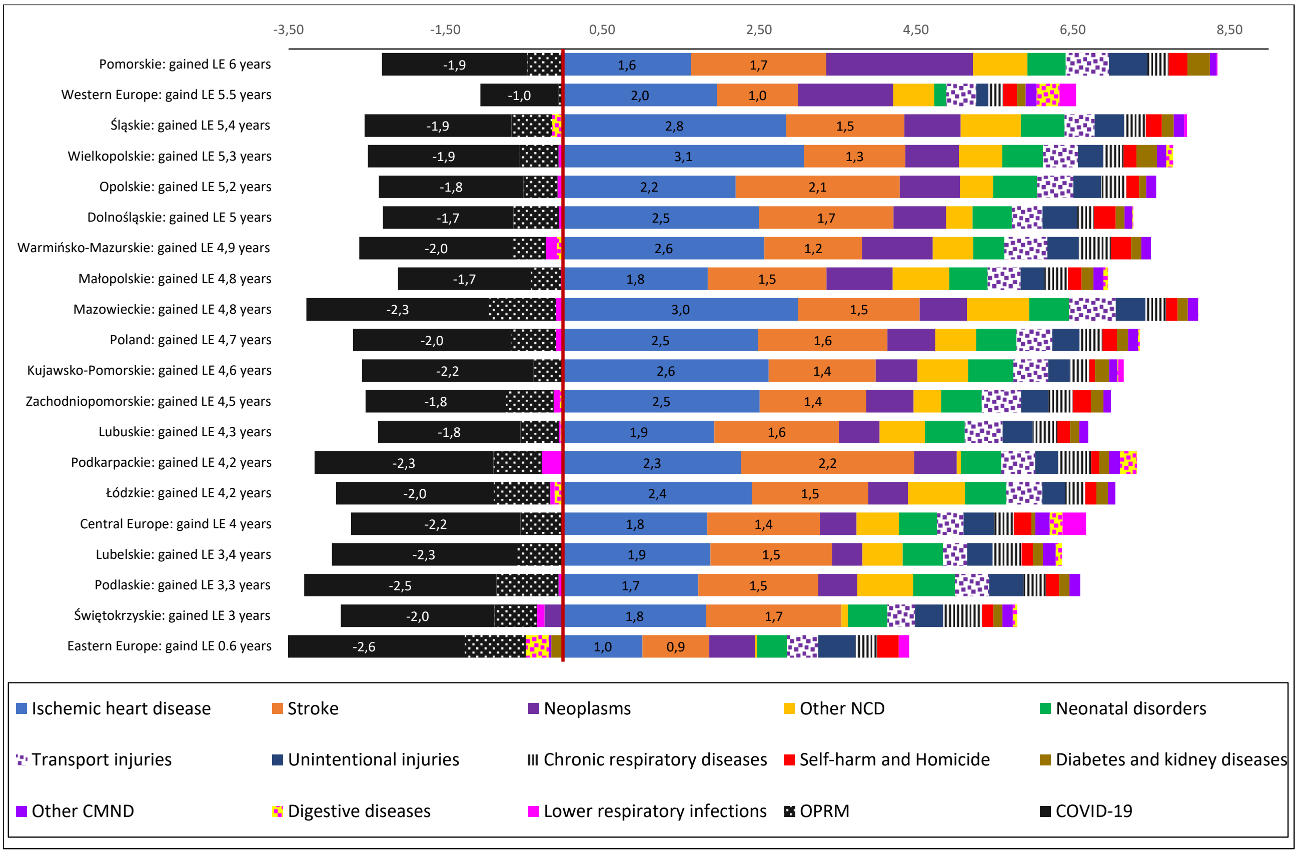

Table S1a. MEDLINE database via Ovid (search date 08/09/2023).

Step	Search	Number of records
1	exp Poland/	53,218
2	Poland.af.	502,102
3	Polish population.af.	2,143
4	Polish citizens.af.	84
5	1 or 2 or 3 or 4	502,131
6	exp "Global Burden of Disease"/	1,972
7	Global Burden of Disease.af.	5,799
8	Global Burden of Diseases.af.	667
9	Disease Global Burden.af.	3
10	Disease Global Burdens.af.	0
11	Global Disease Burden.af.	936
12	Global Disease Burdens.af.	22
13	Disease Burden.af.	22,852
14	Disease Burdens.af.	732
15	Burden of disease.af.	16,371
16	Burden of diseases.af.	1,329
17	Global Burden of Disease Study.af.	1,922
18	6 or 7 or 8 or 9 or 10 or 11 or 12 or 13 or 14 or 15 or 16 or 17	38,453
19	exp Disability-Adjusted Life Years/	202
20	Disability Adjusted Life Years.af.	4,651
21	Disability-Adjusted Life Year.af.	1,100
22	DALYs.af.	3,533
23	DALY.af.	16,665
24	Years Lived With Disability.af.	1,139
25	Year Lived With Disability.af.	12
26	exp Life Expectancy/	40,413
27	Life Expectancy.af.	49,059
28	Life Expectancies.af.	1,817
29	Life Extension.af.	1,455
30	Years of life lost.af.	2,427
31	Year of life lost.af.	35
32	Years of Potential Life Lost.af.	605
33	Year of Potential Life Lost.af.	4
34	Years Lost.af.	997
35	YLLs.af.	428
36	YLL.af.	986
37	19 or 20 or 21 or 22 or 23 or 24 or 25 or 26 or 27 or 28 or 29 or 30 or 31 or 32 or 33 or 34 or 35 or 36	91,214
38	5 and 18 and 37	67

Table S1b. Embase via Ovid (search date 08/09/2023).

Step	Search	Number of records
1	exp Poland/	56,171
2	Poland.af.	706,619
3	Polish population.af.	5,961
4	Polish citizens.af.	207
5	1 or 2 or 3 or 4	708,069
6	exp global disease burden/	6,191
7	Global Burden of Disease.af.	61,539
8	Global Burden of Diseases.af.	3,711
9	Disease Global Burden.af.	443
10	Disease Global Burdens.af.	0
11	Global Disease Burden.af.	10,752
12	Global Disease Burdens.af.	77
13	Disease Burden.af.	149,978
14	Disease Burdens.af.	3,141
15	Burden of disease.af.	123,040
16	Burden of diseases.af.	8,527
17	Global Burden of Disease Study.af.	40,891
18	6 or 7 or 8 or 9 or 10 or 11 or 12 or 13 or 14 or 15 or 16 or 17	253,813
19	exp disability-adjusted life year/	4,546
20	Disability Adjusted Life Years.af.	21,485
21	Disability-Adjusted Life Year.af.	7,195
22	DALYs.af.	12,926
23	DALY.af.	109,511
24	Years Lived With Disability.af.	11,000
25	Year Lived With Disability.af.	55
26	exp life expectancy/	61,182
27	Life Expectancy.af.	235,164
28	Life Expectancies.af.	10,696
29	Life Extension.af.	7,847
30	exp "years of potential life lost"/	270
31	Years of life lost.af.	10,337
32	Year of life lost.af.	183
33	Years of Potential Life Lost.af.	3,941
34	Year of Potential Life Lost.af.	26
35	Years Lost.af.	7,677
36	YLLs.af.	1,056
37	YLL.af.	3,070
38	19 or 20 or 21 or 22 or 23 or 24 or 25 or 26 or 27 or 28 or 29 or 30 or 31 or 32 or 33 or 34 or 35 or 36 or 37	385,452
39	5 and 18 and 38	1,516

Table S1c. Cochrane Library (search date 08/09/2023).

Step	Search	Number of records
#1	MeSH descriptor: [Poland] explode all trees	663
#2	Poland	7248
#3	"Polish population"	33
#4	"Polish citizens"	1
#5	#1 or #2 or #3	7255
#6	MeSH descriptor: [Global Burden of Disease] explode all trees	8
#7	"Global Burden of Disease"	449
#8	"Global Burden of Diseases"	212
#9	"Disease Global Burden"	1
#10	"Disease Global Burdens"	0
#11	"Global Disease Burden"	118
#12	"Global Disease Burdens"	1
#13	"Disease Burden"	2616
#14	"Disease Burdens"	28
#15	"Burden of disease"	1394
#16	"Burden of diseases"	408
#17	"Global Burden of Disease Study"	142
#18	#6 or #7 or #8 or #9 or #10 or #11 or #12 or #13 or #14 or #15 or #16 or #17	3870
#19	MeSH descriptor: [Disability-Adjusted Life Years] explode all trees	3
#20	"Disability Adjusted Life Years"	436
#21	"Disability-Adjusted Life Year"	162
#22	DALYs	265
#23	DALY	1381
#24	"Years Lived With Disability"	225
#25	"Year Lived With Disability"	3
#26	MeSH descriptor: [Life Expectancy] explode all trees	2909
#27	"Life Expectancy"	6790
#28	"Life Expectancies"	81
#29	"Life Extension"	48
#30	"Years of life lost"	63
#31	"Year of life lost"	3
#32	"Years Lost"	94
#33	YLLs	7
#34	YLL	21
#35	#19 or #20 or #21 or #22 or #23 or #24 or #25 or #26 or #27 or #28 or #29 or #30 or #31 or #32 or #33 or #34	11311
#38	#5 and #18 and #35	15

Figure S11. GBD studies including Poland results or context

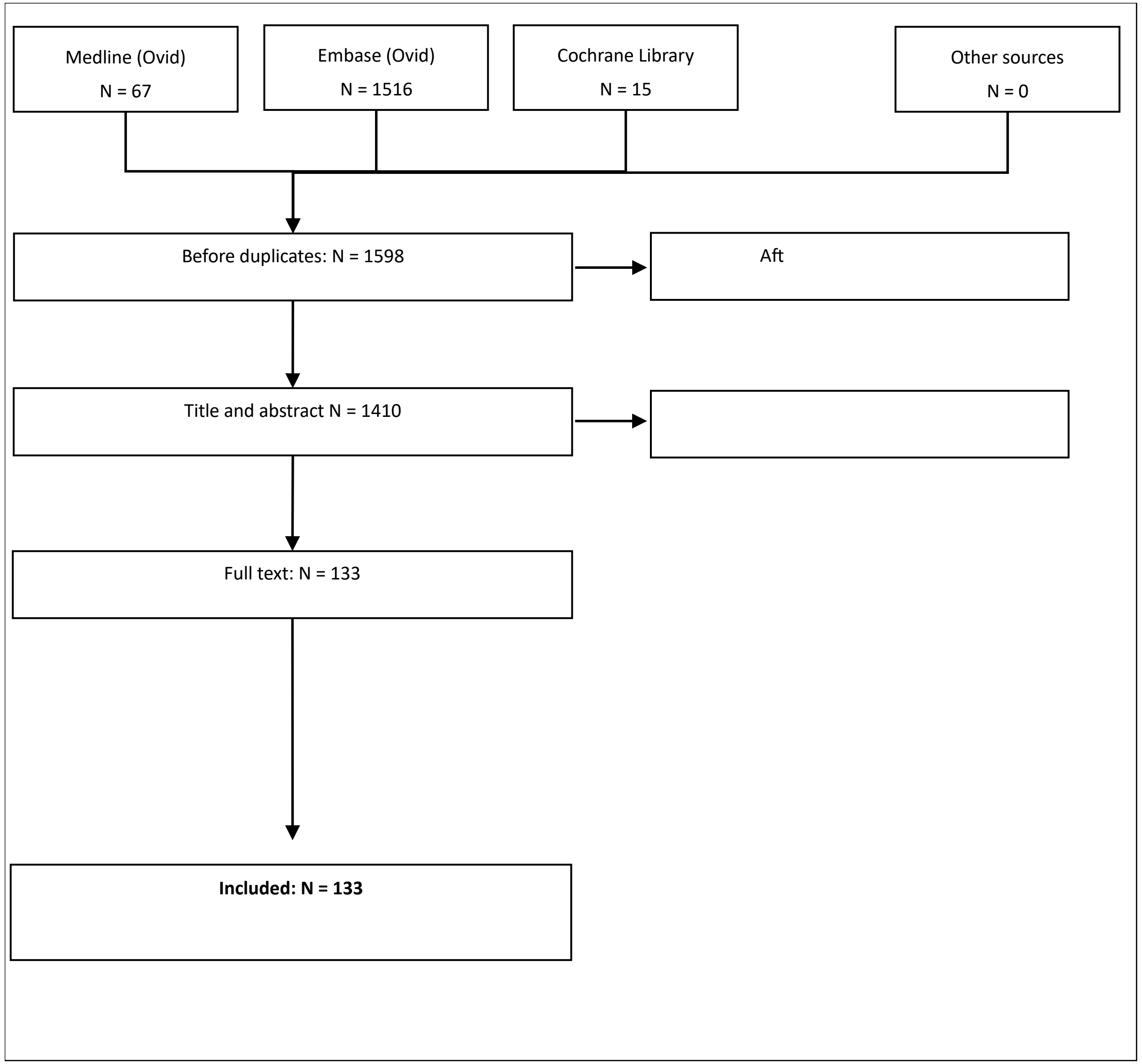

Figure S12. Regional gross domestic product (PPS per inhabitant) by NUTS 2 region

Geopolitical entity (reporting)
/ Time: 2023
Time frequency: Annual
Unit of measure: Purchasing power standard (PPS, EU27 from 2020), per inhabitant

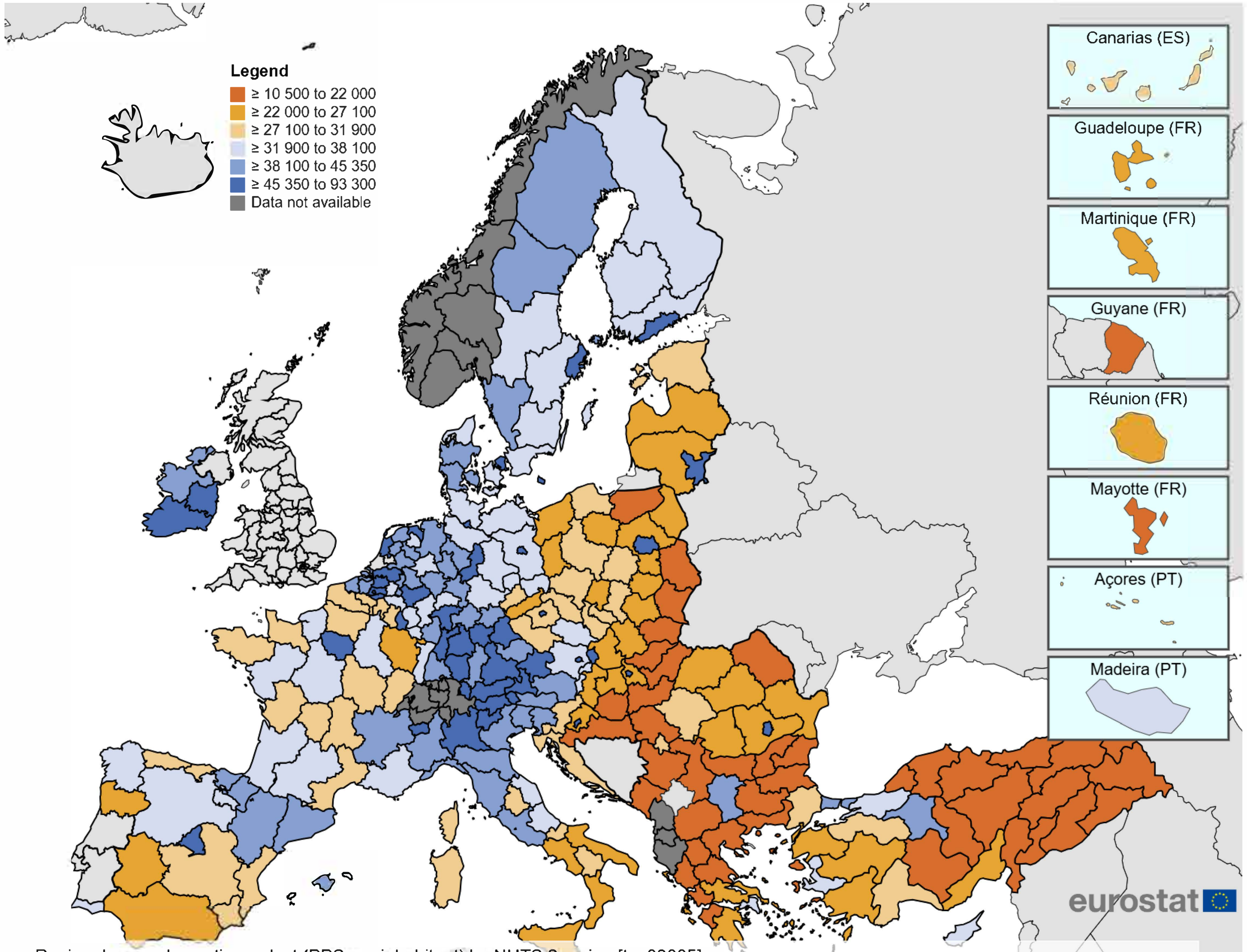

Regional gross domestic product (PPS per inhabitant) by NUTS 2 region [tgs00005]
Source of data: Eurostat - Last updated date: 18/03/2025 23:00
Disclaimer This map has been created automatically by ESTAT/EC software according to external user specifications for which ESTAT/EC is not responsible. Map included.
General disclaimer of the EC website: https://ec.europa.eu/info/legal-notice_en
Built with Vega Administration boundaries: ©EuroGeographics ©UN-FAO ©Turkstat Cartography: Eurostat - GISCO - 2024

Figure S13. Life expectancy, with population size (millions) 2023

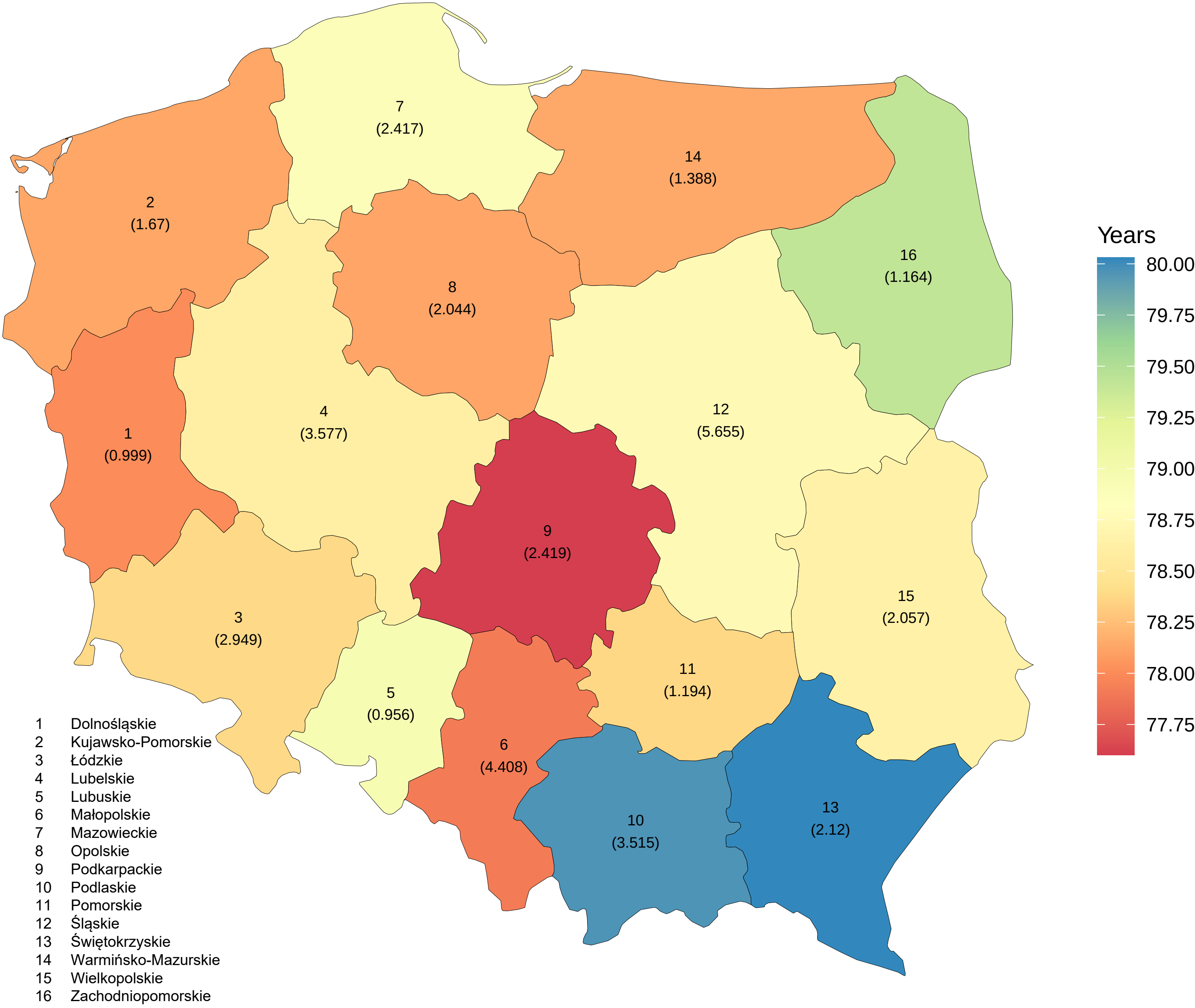

Table S2. Percentage change in risk-attributable burden by DALYs in Poland between 1990 and 2023

Risk Factor	Year	Western Europe	Eastern Europe	Central Europe	Poland	Dolnoslaskie	Kujawsko-Pomorskie	Lodzkie	Lubelskie	Lubuskie
Smoking	1990	2,988.5 (2,489.5 to 3,498.9)	3,643.3 (3,080.0 to 4,276.5)	4,483.1 (3,652.7 to 5,317.7)	5,478.3 (4,553.1 to 6,330.3)	5,980.9 (4,991.7 to 6,921.8)	5,978.9 (4,951.4 to 6,909.0)	5,368.9 (4,422.5 to 6,288.7)	5,193.4 (4,307.2 to 6,030.8)	5,330.8 (4,381.0 to 6,279.5)
	2023	1,412.7 (1,102.2 to 1,729.0)	2,686.0 (2,222.4 to 3,149.8)	2,366.9 (1,921.0 to 2,846.1)	2,269.4 (1,853.3 to 2,712.3)	2,538.2 (2,072.5 to 3,065.6)	2,745.5 (2,257.9 to 3,269.2)	2,220.2 (1,788.6 to 2,719.0)	2,298.2 (1,886.3 to 2,793.6)	2,246.8 (1,752.6 to 2,776.9)
	% change from 1990	-52.7 (-57.5 to -48.0)	-26.3 (-35.5 to -15.5)	-47.2 (-54.1 to -39.4)	-58.6 (-63.2 to -54.4)	-57.6 (-63.0 to -52.4)	-54.1 (-59.8 to -48.9)	-58.6 (-64.0 to -53.5)	-55.7 (-60.7 to -50.8)	-57.9 (-64.3 to -51.4)
High systolic blood pressure	1990	2,928.0 (2,469.8 to 3,353.4)	6,544.4 (5,388.5 to 7,636.1)	6,217.7 (5,233.5 to 7,081.5)	5,723.8 (4,759.8 to 6,578.3)	5,984.8 (4,913.8 to 6,956.3)	5,653.3 (4,581.8 to 6,531.4)	6,015.7 (5,035.3 to 6,814.9)	5,545.1 (4,551.6 to 6,456.1)	5,874.2 (4,741.4 to 6,864.2)
	2023	1,071.5 (868.8 to 1,235.4)	4,271.5 (3,391.8 to 4,962.4)	2,844.3 (2,379.2 to 3,199.0)	2,053.7 (1,657.2 to 2,362.4)	2,246.6 (1,754.3 to 2,670.6)	2,069.8 (1,656.6 to 2,405.3)	2,047.5 (1,666.0 to 2,374.0)	2,274.1 (1,819.1 to 2,651.3)	2,606.8 (2,063.6 to 3,043.8)
	% change from 1990	-63.4 (-67.2 to -59.6)	-34.7 (-45.7 to -20.5)	-54.3 (-57.9 to -49.2)	-64.1 (-67.9 to -58.9)	-62.5 (-66.9 to -55.9)	-63.4 (-67.5 to -58.0)	-66.0 (-70.8 to -60.8)	-59.0 (-63.6 to -52.3)	-55.6 (-60.9 to -49.6)
High body-mass index	1990	1,381.9 (560.7 to 2,203.1)	1,942.9 (775.0 to 3,163.9)	2,040.8 (899.4 to 3,246.1)	2,226.4 (966.8 to 3,512.2)	2,321.1 (986.8 to 3,815.1)	2,433.4 (1,091.4 to 3,774.1)	2,175.4 (921.9 to 3,483.8)	2,068.0 (912.1 to 3,227.6)	2,207.9 (967.0 to 3,481.1)
	2023	1,192.1 (485.5 to 1,832.9)	2,082.5 (886.9 to 3,257.2)	1,872.0 (882.2 to 2,772.3)	1,923.4 (839.0 to 2,907.8)	1,994.2 (853.5 to 3,078.8)	2,063.4 (907.9 to 3,123.8)	1,926.1 (840.4 to 2,987.3)	1,991.2 (871.5 to 3,028.4)	2,070.4 (919.4 to 3,145.7)
	% change from 1990	-13.7 (-29.6 to 4.3)	7.2 (-12.5 to 28.4)	-8.3 (-26.0 to 12.5)	-13.6 (-32.3 to 6.0)	-14.1 (-32.6 to 8.3)	-15.2 (-31.2 to 1.7)	-11.5 (-33.5 to 12.5)	-3.7 (-24.8 to 14.7)	-6.2 (-25.4 to 14.2)
High fasting plasma glucose	1990	1,323.7 (1,113.0 to 1,580.6)	1,175.1 (865.6 to 1,577.7)	1,958.4 (1,544.2 to 2,669.6)	2,443.4 (1,998.0 to 3,131.8)	1,960.2 (1,611.5 to 2,450.6)	2,507.8 (2,047.1 to 3,255.6)	2,617.0 (2,199.5 to 3,209.0)	2,228.5 (1,821.4 to 2,911.9)	2,470.6 (1,968.6 to 3,206.5)
	2023	974.2 (815.3 to 1,134.4)	1,367.5 (1,123.0 to 1,743.7)	1,692.0 (1,384.5 to 2,089.1)	1,784.1 (1,512.3 to 2,114.8)	1,652.5 (1,407.9 to 1,925.8)	1,834.6 (1,542.0 to 2,194.9)	1,901.5 (1,614.1 to 2,272.5)	1,858.2 (1,558.0 to 2,185.3)	1,912.5 (1,628.6 to 2,273.4)
	% change from 1990	-26.4 (-37.5 to -15.0)	16.4 (-11.8 to 51.9)	-13.6 (-36.0 to 10.9)	-27.0 (-40.9 to -14.4)	-15.7 (-30.7 to -1.0)	-26.8 (-41.0 to -12.4)	-27.3 (-39.8 to -14.9)	-16.6 (-34.3 to 0.0)	-22.6 (-39.6 to -6.5)
High alcohol use	1990	1,134.3 (934.1 to 1,441.4)	2,105.0 (1,759.6 to 2,507.6)	1,394.7 (1,130.3 to 1,800.1)	1,062.4 (825.8 to 1,466.2)	1,058.2 (796.9 to 1,496.7)	946.9 (715.4 to 1,367.5)	1,263.7 (989.3 to 1,724.8)	1,014.3 (782.5 to 1,370.4)	1,098.9 (863.4 to 1,526.6)
	2023	746.0 (609.7 to 904.4)	2,041.7 (1,836.2 to 2,356.7)	1,230.3 (1,056.2 to 1,462.9)	1,436.9 (1,239.7 to 1,678.4)	1,411.1 (1,185.9 to 1,685.3)	1,341.8 (1,124.0 to 1,591.1)	1,797.8 (1,551.0 to 2,104.4)	1,394.6 (1,165.6 to 1,670.7)	1,263.8 (1,047.8 to 1,609.7)
	% change from 1990	-34.2 (-40.7 to -28.5)	-3.0 (-10.4 to 6.9)	-11.8 (-21.0 to -3.0)	35.2 (3.5 to 65.1)	33.4 (4.4 to 66.0)	41.7 (6.3 to 75.7)	42.3 (6.7 to 74.4)	37.5 (14.9 to 66.4)	15.0 (-4.8 to 35.6)
High LDL cholesterol	1990	1,566.1 (1,025.3 to 2,100.9)	3,749.4 (2,354.1 to 5,197.7)	2,907.5 (1,841.9 to 4,038.5)	2,974.8 (1,943.3 to 4,007.4)	3,176.9 (2,063.4 to 4,313.7)	2,988.2 (1,965.4 to 3,993.3)	3,280.9 (2,199.5 to 4,400.4)	2,608.8 (1,693.0 to 3,594.6)	2,992.6 (1,957.4 to 4,088.3)
	2023	463.7 (315.4 to 631.8)	2,252.4 (1,493.1 to 3,046.2)	1,127.1 (732.4 to 1,573.8)	865.5 (557.6 to 1,196.2)	1,076.7 (672.2 to 1,490.5)	962.0 (633.6 to 1,298.7)	743.9 (467.0 to 1,040.4)	1,000.2 (679.8 to 1,387.8)	1,316.5 (897.7 to 1,759.4)
	% change from 1990	-70.4 (-74.0 to -67.4)	-39.9 (-48.7 to -28.4)	-61.2 (-66.2 to -56.1)	-70.9 (-74.2 to -67.7)	-66.1 (-71.6 to -60.2)	-67.8 (-71.2 to -64.0)	-77.3 (-80.2 to -74.3)	-61.7 (-66.1 to -56.3)	-56.0 (-61.7 to -49.5)
Particulate matter pollution	1990	1,364.3 (740.5 to 2,029.9)	3,551.6 (2,075.2 to 4,922.6)	3,824.3 (2,686.6 to 4,941.5)	3,778.1 (2,686.1 to 4,849.0)	3,847.0 (2,641.8 to 5,021.6)	3,788.5 (2,608.2 to 4,879.5)	4,266.2 (3,092.6 to 5,441.0)	3,506.6 (2,467.8 to 4,522.1)	3,517.1 (2,420.1 to 4,632.4)
	2023	321.7 (202.3 to 459.8)	937.6 (645.7 to 1,257.8)	1,033.3 (773.0 to 1,347.2)	834.8 (619.9 to 1,091.9)	849.6 (619.7 to 1,105.0)	784.6 (576.3 to 1,047.1)	1,046.7 (804.6 to 1,344.3)	880.4 (646.3 to 1,143.8)	665.4 (449.0 to 934.7)
	% change from 1990	-76.4 (-85.2 to -60.0)	-73.6 (-81.8 to -61.9)	-73.0 (-79.8 to -64.9)	-77.9 (-83.4 to -70.9)	-77.9 (-83.8 to -70.5)	-79.3 (-85.0 to -72.0)	-75.5 (-80.8 to -68.0)	-74.9 (-81.3 to -67.0)	-81.1 (-87.4 to -73.9)
Lead exposure	1990	660.5 (551.1 to 778.9)	1,522.6 (1,269.8 to 1,803.5)	1,277.4 (1,052.5 to 1,524.8)	1,490.0 (1,229.6 to 1,754.9)	1,577.8 (1,279.8 to 1,927.6)	1,490.0 (1,234.6 to 1,804.7)	1,513.7 (1,241.8 to 1,822.6)	1,376.3 (1,093.0 to 1,710.0)	1,484.1 (1,204.5 to 1,864.1)
	2023	240.4 (195.6 to 291.9)	1,259.4 (1,076.8 to 1,451.3)	655.0 (539.7 to 776.0)	602.3 (488.2 to 717.4)	753.6 (630.9 to 885.2)	624.3 (508.9 to 751.8)	537.4 (429.0 to 666.7)	770.8 (645.9 to 921.7)	791.9 (656.0 to 951.6)
	% change from 1990	-63.6 (-66.5 to -61.2)	-17.3 (-22.8 to -12.1)	-48.7 (-51.8 to -46.2)	-59.6 (-62.6 to -56.1)	-52.2 (-56.4 to -47.5)	-58.1 (-62.0 to -53.8)	-64.5 (-67.9 to -60.6)	-44.0 (-50.0 to -37.8)	-46.6 (-51.5 to -41.0)
Kidney dysfunction	1990	659.2 (536.1 to 787.2)	1,611.6 (1,254.4 to 2,007.3)	1,091.9 (878.3 to 1,316.8)	1,192.4 (956.8 to 1,449.3)	1,272.9 (1,034.2 to 1,566.0)	1,194.1 (971.5 to 1,458.7)	1,188.8 (938.9 to 1,457.5)	1,123.4 (911.9 to 1,349.4)	1,233.4 (999.0 to 1,479.5)
	2023	382.7 (328.8 to 443.9)	1,079.5 (839.9 to 1,335.1)	603.1 (503.9 to 713.4)	493.0 (407.8 to 589.5)	525.2 (423.5 to 646.9)	492.4 (406.3 to 590.3)	506.9 (422.1 to 604.5)	531.2 (428.3 to 644.9)	588.6 (477.2 to 700.4)
	% change from 1990	-41.9 (-46.8 to -36.1)	-33.0 (-36.1 to -30.0)	-44.8 (-47.3 to -42.0)	-58.7 (-60.7 to -56.1)	-58.7 (-61.9 to -55.2)	-58.8 (-61.7 to -55.6)	-57.4 (-60.4 to -53.6)	-52.7 (-56.8 to -48.5)	-52.3 (-56.1 to -48.3)
Diet high in sodium	1990	215.7 (1.3 to 800.1)	610.7 (12.7 to 1,974.4)	1,544.6 (444.3 to 2,845.8)	948.6 (90.3 to 2,244.3)	995.2 (92.0 to 2,370.1)	925.1 (83.0 to 2,184.8)	952.1 (90.2 to 2,265.0)	927.6 (91.6 to 2,177.6)	967.0 (86.4 to 2,257.9)
	2023	92.4 (0.7 to 313.0)	413.4 (12.9 to 1,228.3)	691.7 (160.2 to 1,325.5)	427.2 (47.2 to 964.3)	470.6 (48.0 to 1,073.6)	432.8 (45.2 to 996.1)	389.6 (40.8 to 901.5)	470.3 (51.8 to 1,062.5)	535.0 (57.5 to 1,248.2)
	% change from 1990	-57.2 (-93.9 to 345.7)	-32.3 (-85.4 to 430.5)	-55.2 (-77.5 to -29.0)	-55.0 (-83.5 to 44.4)	-52.7 (-83.7 to 52.2)	-53.2 (-82.9 to 60.2)	-59.1 (-85.5 to 41.2)	-49.3 (-82.5 to 62.4)	-44.7 (-79.8 to 94.9)

Risk Factor	Year	Malopolskie	Mazowieckie	Opolskie	Podkarpackie	Podlaskie	Pomorskie	Slaskie	Swietokrzyskie	Warminsko-Mazurskie	Wielkopolskie	Zachodniopomorskie
Smoking	1990	4,969.9 (4,149.2 to 5,824.6)	5,809.0 (4,780.1 to 6,724.2)	5,166.2 (4,279.3 to 6,063.5)	4,719.9 (3,972.4 to 5,478.2)	4,413.7 (3,677.8 to 5,169.6)	5,585.1 (4,659.4 to 6,476.4)	5,747.2 (4,780.6 to 6,640.3)	4,317.8 (3,509.0 to 5,092.7)	5,983.8 (5,063.8 to 6,938.4)	5,771.8 (4,791.7 to 6,654.9)	5,976.1 (4,997.2 to 6,886.5)
	2023	2,094.8 (1,640.5 to 2,554.9)	2,378.0 (1,915.4 to 2,843.9)	2,058.5 (1,612.9 to 2,497.5)	1,897.3 (1,531.5 to 2,294.3)	1,974.4 (1,569.8 to 2,389.6)	2,140.7 (1,708.5 to 2,587.8)	2,141.5 (1,696.6 to 2,597.7)	2,081.7 (1,678.4 to 2,540.2)	2,361.6 (1,931.4 to 2,824.3)	2,335.4 (1,897.9 to 2,802.1)	2,529.7 (2,044.1 to 3,019.1)
	% change from 1990	-57.8 (-63.3 to -52.7)	-59.1 (-63.7 to -54.5)	-60.2 (-66.7 to -54.0)	-59.8 (-65.3 to -54.8)	-55.3 (-61.4 to -48.9)	-61.7 (-66.3 to -56.9)	-62.7 (-67.8 to -58.3)	-51.8 (-58.5 to -45.3)	-60.5 (-65.6 to -55.9)	-59.5 (-64.1 to -54.0)	-57.7 (-62.5 to -52.8)
High systolic blood pressure	1990	4,995.5 (4,060.0 to 5,821.8)	6,293.8 (5,285.2 to 7,159.2)	5,731.3 (4,606.8 to 6,715.9)	5,508.6 (4,521.6 to 6,325.0)	4,598.0 (3,766.2 to 5,382.3)	5,565.2 (4,500.3 to 6,479.8)	6,412.6 (5,295.9 to 7,412.9)	4,822.3 (3,950.0 to 5,633.9)	5,517.9 (4,506.0 to 6,456.4)	5,977.9 (4,853.1 to 6,996.5)	5,538.6 (4,490.7 to 6,453.9)
	2023	1,981.5 (1,561.2 to 2,305.9)	1,835.3 (1,479.7 to 2,163.2)	2,048.6 (1,680.1 to 2,392.4)	2,072.1 (1,744.1 to 2,350.8)	1,927.4 (1,552.0 to 2,229.1)	1,872.0 (1,497.6 to 2,200.1)	2,212.0 (1,773.0 to 2,547.9)	2,363.2 (1,913.8 to 2,749.0)	1,825.4 (1,469.0 to 2,137.2)	1,893.4 (1,514.7 to 2,206.8)	2,069.1 (1,646.3 to 2,411.4)
	% change from 1990	-60.3 (-64.7 to -54.3)	-70.8 (-75.1 to -65.5)	-64.3 (-68.6 to -58.4)	-62.4 (-66.6 to -55.5)	-58.1 (-63.3 to -51.1)	-66.4 (-70.4 to -60.9)	-65.5 (-69.8 to -59.9)	-51.0 (-58.8 to -39.7)	-66.9 (-71.3 to -61.7)	-68.3 (-71.9 to -63.3)	-62.6 (-67.4 to -57.7)
High body-mass index	1990	2,029.1 (912.7 to 3,152.6)	2,198.2 (945.6 to 3,500.9)	2,236.9 (973.4 to 3,503.7)	2,114.1 (980.7 to 3,272.2)	2,179.0 (948.5 to 3,363.7)	2,272.1 (981.4 to 3,592.6)	2,437.5 (1,084.3 to 3,886.2)	1,855.9 (800.1 to 2,968.7)	1,995.9 (858.9 to 3,255.0)	2,505.5 (1,078.1 to 3,945.1)	2,308.7 (988.8 to 3,666.0)
	2023	1,904.6 (812.4 to 2,880.9)	1,839.5 (781.2 to 2,873.7)	1,971.5 (888.4 to 2,966.8)	1,979.5 (921.2 to 2,960.7)	1,940.2 (876.0 to 2,915.9)	1,891.1 (817.5 to 2,802.8)	1,923.6 (886.4 to 2,889.2)	1,950.4 (854.1 to 2,954.5)	1,738.9 (744.0 to 2,666.1)	1,883.5 (814.9 to 2,867.9)	1,910.6 (829.3 to 2,897.1)
	% change from 1990	-6.1 (-26.2 to 13.5)	-16.3 (-38.3 to 8.1)	-11.9 (-28.3 to 6.2)	-6.4 (-26.4 to 20.7)	-11.0 (-27.2 to 9.3)	-16.8 (-32.8 to -0.3)	-21.1 (-39.2 to 3.2)	5.1 (-17.7 to 30.3)	-12.9 (-32.6 to 5.6)	-24.8 (-40.7 to -5.7)	-17.2 (-33.7 to 4.5)
High fasting plasma glucose	1990	2,179.9 (1,801.8 to 2,729.6)	2,605.8 (2,135.7 to 3,327.2)	2,548.1 (2,092.0 to 3,245.4)	2,327.0 (1,907.3 to 2,957.7)	2,362.5 (1,915.7 to 2,969.2)	2,749.4 (2,298.2 to 3,411.3)	2,674.9 (2,166.4 to 3,570.6)	1,932.3 (1,590.0 to 2,438.6)	2,543.5 (2,073.3 to 3,272.0)	2,777.2 (2,279.0 to 3,604.7)	2,387.8 (1,887.5 to 3,182.2)
	2023	1,729.3 (1,438.1 to 2,084.8)	1,773.3 (1,498.6 to 2,129.8)	1,861.7 (1,553.0 to 2,212.7)	1,747.4 (1,453.3 to 2,110.8)	1,667.9 (1,400.4 to 1,999.3)	1,870.0 (1,610.1 to 2,223.6)	1,799.6 (1,517.3 to 2,122.9)	1,712.0 (1,485.7 to 1,983.0)	1,788.9 (1,508.1 to 2,121.9)	1,790.6 (1,510.7 to 2,108.5)	1,737.5 (1,476.4 to 2,043.4)
	% change from 1990	-20.7 (-36.4 to -5.8)	-31.9 (-45.7 to -20.1)	-26.9 (-41.5 to -13.9)	-24.9 (-38.4 to -11.7)	-29.4 (-41.4 to -16.0)	-32.0 (-44.4 to -20.1)	-32.7 (-47.1 to -18.2)	-11.4 (-26.0 to 2.5)	-29.7 (-42.8 to -17.6)	-35.5 (-48.7 to -23.9)	-27.2 (-41.5 to -11.0)
High alcohol use	1990	841.3 (621.9 to 1,188.4)	1,236.9 (958.2 to 1,663.5)	903.8 (662.0 to 1,326.2)	839.0 (624.0 to 1,209.9)	1,171.7 (933.3 to 1,538.9)	1,069.8 (813.9 to 1,489.6)	1,120.5 (839.5 to 1,601.0)	927.6 (720.3 to 1,267.2)	1,159.5 (909.5 to 1,547.4)	1,026.4 (775.5 to 1,484.3)	1,081.5 (846.5 to 1,471.4)
	2023	1,157.2 (971.1 to 1,404.5)	1,451.1 (1,252.9 to 1,706.4)	1,250.3 (1,048.2 to 1,494.1)	1,214.7 (1,017.0 to 1,439.2)	1,532.0 (1,309.0 to 1,797.7)	1,501.2 (1,281.5 to 1,751.6)	1,611.9 (1,366.3 to 1,932.4)	1,513.7 (1,276.2 to 1,765.9)	1,620.0 (1,399.9 to 1,866.6)	1,342.0 (1,139.9 to 1,598.1)	1,547.7 (1,339.4 to 1,799.6)
	% change from 1990	37.6 (4.3 to 73.2)	17.3 (-7.8 to 43.0)	38.3 (2.9 to 74.2)	44.8 (5.4 to 79.5)	30.7 (9.1 to 57.6)	40.3 (8.7 to 75.2)	43.8 (1.1 to 82.5)	63.2 (24.8 to 99.7)	39.7 (12.6 to 70.4)	30.8 (-4.8 to 63.7)	43.1 (8.8 to 75.0)
High LDL cholesterol	1990	2,674.4 (1,702.0 to 3,625.0)	3,048.5 (1,909.4 to 4,210.5)	2,883.5 (1,842.3 to 3,895.2)	2,661.9 (1,710.8 to 3,636.0)	2,264.4 (1,463.3 to 3,074.6)	2,808.1 (1,825.8 to 3,742.7)	3,510.2 (2,338.5 to 4,666.1)	2,428.2 (1,549.3 to 3,308.5)	2,861.0 (1,823.6 to 3,886.3)	3,215.8 (2,142.6 to 4,309.8)	2,970.9 (1,934.8 to 4,009.1)
	2023	861.4 (545.9 to 1,201.5)	725.0 (446.1 to 1,016.0)	866.4 (573.2 to 1,185.0)	776.7 (507.4 to 1,059.2)	781.5 (520.4 to 1,085.3)	837.1 (559.3 to 1,161.9)	897.1 (554.2 to 1,252.7)	910.9 (577.2 to 1,274.8)	767.0 (491.1 to 1,064.2)	788.5 (494.5 to 1,109.6)	898.0 (596.4 to 1,224.5)
	% change from 1990	-67.8 (-72.1 to -63.4)	-76.2 (-79.5 to -72.5)	-70.0 (-73.8 to -65.5)	-70.8 (-74.4 to -66.9)	-65.5 (-70.3 to -59.9)	-70.2 (-73.7 to -66.4)	-74.4 (-78.2 to -70.9)	-62.5 (-68.0 to -56.6)	-73.2 (-76.7 to -69.6)	-75.5 (-78.9 to -72.2)	-69.8 (-73.3 to -65.9)
Particulate matter pollution	1990	3,664.4 (2,668.3 to 4,655.9)	3,818.7 (2,583.4 to 5,036.2)	3,861.8 (2,752.3 to 4,947.9)	3,465.8 (2,584.4 to 4,525.2)	3,051.7 (2,153.0 to 3,897.5)	3,370.1 (2,251.5 to 4,391.9)	4,661.9 (3,393.9 to 5,741.1)	3,342.0 (2,393.0 to 4,277.1)	3,387.9 (2,295.4 to 4,433.3)	3,889.4 (2,684.9 to 4,986.8)	3,255.1 (2,177.5 to 4,285.5)
	2023	1,017.5 (793.9 to 1,287.4)	770.9 (553.5 to 1,028.0)	873.3 (651.4 to 1,138.8)	752.3 (567.5 to 978.0)	655.1 (458.3 to 862.0)	572.1 (366.4 to 792.7)	1,136.1 (885.8 to 1,418.3)	909.9 (672.2 to 1,193.7)	606.2 (411.5 to 819.3)	773.5 (570.3 to 1,003.6)	451.3 (263.0 to 684.6)
	% change from 1990	-72.2 (-78.2 to -64.1)	-79.8 (-85.8 to -71.9)	-77.4 (-82.8 to -70.3)	-78.3 (-84.0 to -71.8)	-78.5 (-84.3 to -71.8)	-83.0 (-88.7 to -77.0)	-75.6 (-80.7 to -69.3)	-72.8 (-79.4 to -63.8)	-82.1 (-87.7 to -74.4)	-80.1 (-85.3 to -73.4)	-86.1 (-91.5 to -79.7)
Lead exposure	1990	1,359.1 (1,115.9 to 1,668.1)	1,557.1 (1,279.9 to 1,878.9)	1,380.7 (1,130.1 to 1,724.8)	1,423.3 (1,159.2 to 1,723.7)	1,197.7 (982.3 to 1,451.9)	1,486.3 (1,208.7 to 1,775.1)	1,712.7 (1,420.7 to 2,087.7)	1,223.2 (1,002.9 to 1,505.6)	1,456.1 (1,210.6 to 1,726.8)	1,637.5 (1,355.9 to 1,991.0)	1,492.4 (1,227.7 to 1,803.3)
	2023	583.6 (475.3 to 706.9)	504.8 (400.1 to 620.0)	602.4 (495.0 to 739.3)	583.8 (457.1 to 721.4)	613.2 (499.3 to 751.3)	599.4 (491.6 to 711.8)	611.0 (476.9 to 776.1)	616.8 (490.7 to 756.8)	536.0 (434.5 to 659.1)	522.3 (411.5 to 664.7)	617.2 (496.5 to 763.4)
	% change from 1990	-57.1 (-60.5 to -52.5)	-67.6 (-70.3 to -64.1)	-56.4 (-59.9 to -51.3)	-59.0 (-62.7 to -54.1)	-48.8 (-53.2 to -43.6)	-59.7 (-63.0 to -55.7)	-64.3 (-67.8 to -60.7)	-49.6 (-54.6 to -43.9)	-63.2 (-67.1 to -59.3)	-68.1 (-71.5 to -64.4)	-58.6 (-62.4 to -54.5)
Kidney dysfunction	1990	1,063.2 (853.3 to 1,283.2)	1,301.6 (1,030.8 to 1,596.0)	1,156.9 (916.5 to 1,414.9)	1,164.6 (945.7 to 1,391.5)	993.3 (806.3 to 1,210.9)	1,205.9 (962.0 to 1,476.5)	1,326.5 (1,064.6 to 1,612.8)	1,018.1 (825.4 to 1,221.9)	1,196.1 (956.5 to 1,442.4)	1,231.2 (988.9 to 1,494.6)	1,174.2 (935.7 to 1,438.0)
	2023	452.4 (369.8 to 546.5)	448.9 (370.9 to 527.2)	496.6 (414.8 to 596.0)	496.1 (418.1 to 584.2)	451.3 (368.7 to 555.8)	502.7 (416.1 to 596.9)	521.0 (431.4 to 633.3)	508.8 (414.0 to 606.4)	451.2 (372.9 to 546.0)	484.4 (404.4 to 571.7)	503.1 (418.1 to 609.3)
	% change from 1990	-57.4 (-60.0 to -54.3)	-65.5 (-67.8 to -62.6)	-57.1 (-60.6 to -51.8)	-57.4 (-60.4 to -53.7)	-54.6 (-58.3 to -50.6)	-58.3 (-61.0 to -54.4)	-60.7 (-63.0 to -57.8)	-50.0 (-54.1 to -46.4)	-62.3 (-65.2 to -59.0)	-60.7 (-63.6 to -57.0)	-57.2 (-60.1 to -53.6)
Diet high in sodium	1990	900.9 (86.4 to 2,125.7)	1,038.5 (105.7 to 2,445.0)	922.0 (82.5 to 2,207.1)	916.0 (92.8 to 2,152.6)	775.0 (77.3 to 1,846.2)	938.8 (86.4 to 2,205.0)	1,052.5 (96.0 to 2,504.8)	802.4 (78.7 to 1,922.8)	922.4 (88.6 to 2,183.6)	992.2 (96.9 to 2,371.6)	905.8 (80.2 to 2,189.6)
	2023	415.3 (47.1 to 957.4)	378.0 (42.1 to 829.0)	430.6 (47.8 to 973.2)	442.0 (54.1 to 938.0)	411.2 (48.5 to 897.8)	394.4 (43.4 to 883.7)	470.4 (52.6 to 1,048.6)	459.8 (52.1 to 1,034.6)	387.9 (45.4 to 876.3)	402.7 (44.5 to 910.7)	436.8 (46.7 to 1,013.2)
	% change from 1990	-53.9 (-82.8 to 40.8)	-63.6 (-87.1 to 12.7)	-53.3 (-82.3 to 70.3)	-51.8 (-81.9 to 63.7)	-46.9 (-80.8 to 71.3)	-58.0 (-84.4 to 35.9)	-55.3 (-82.8 to 50.2)	-42.7 (-78.7 to 78.4)	-57.9 (-84.2 to 32.7)	-59.4 (-84.8 to 30.1)	-51.8 (-81.7 to 62.6)

Table S3. Life expectancy decomposition by causes in Poland, 16 voivodships and European regions between 1990 and 2023

Name of the cause	Location	Value
Ischemic heart disease	Central Europe	1.9
Stroke	Central Europe	1.6
Neoplasms	Central Europe	0.7
Chronic respiratory diseases	Central Europe	0.3
Digestive diseases	Central Europe	0.1
Diabetes and kidney diseases	Central Europe	0.0
Other NCD	Central Europe	0.5
Lower respiratory infections	Central Europe	0.2
COVID-19	Central Europe	-0.1
Neonatal disorders	Central Europe	0.4
Other CMMND	Central Europe	0.2
Self-harm and interpersonal violence	Central Europe	0.2
Transport injuries	Central Europe	0.4
Unintentional injuries	Central Europe	0.3
Ischemic heart disease	Eastern Europe	1.3
Stroke	Eastern Europe	1.1
Neoplasms	Eastern Europe	0.7
Chronic respiratory diseases	Eastern Europe	0.2
Digestive diseases	Eastern Europe	-0.2
Diabetes and kidney diseases	Eastern Europe	-0.1
Other NCD	Eastern Europe	0.0
Lower respiratory infections	Eastern Europe	0.2
COVID-19	Eastern Europe	0.0
Neonatal disorders	Eastern Europe	0.3
Other CMMND	Eastern Europe	0.1
Self-harm and interpersonal violence	Eastern Europe	-0.9
Transport injuries	Eastern Europe	0.4
Unintentional injuries	Eastern Europe	0.5
Ischemic heart disease	Western Europe	1.9
Stroke	Western Europe	1.0
Neoplasms	Western Europe	1.2
Chronic respiratory diseases	Western Europe	0.2
Digestive diseases	Western Europe	0.2
Diabetes and kidney diseases	Western Europe	0.1
Other NCD	Western Europe	0.3
Lower respiratory infections	Western Europe	0.2
COVID-19	Western Europe	-0.1
Neonatal disorders	Western Europe	0.1
Other CMMND	Western Europe	0.1
Self-harm and interpersonal violence	Western Europe	0.1
Transport injuries	Western Europe	0.4
Unintentional injuries	Western Europe	0.0
Ischemic heart disease	Poland	2.5

Stroke	Poland	1.7
Neoplasms	Poland	1.0
Chronic respiratory diseases	Poland	0.3
Digestive diseases	Poland	-0.1
Diabetes and kidney diseases	Poland	0.1
Other NCD	Poland	0.6
Lower respiratory infections	Poland	-0.2
COVID-19	Poland	-0.1
Neonatal disorders	Poland	0.5
Other CMMND	Poland	0.1
Self-harm and interpersonal violence	Poland	0.2
Transport injuries	Poland	0.5
Unintentional injuries	Poland	0.3
Ischemic heart disease	Dolnośląskie	2.2
Stroke	Dolnośląskie	1.9
Neoplasms	Dolnośląskie	1.1
Chronic respiratory diseases	Dolnośląskie	0.3
Digestive diseases	Dolnośląskie	-0.1
Diabetes and kidney diseases	Dolnośląskie	0.1
Other NCD	Dolnośląskie	0.5
Lower respiratory infections	Dolnośląskie	-0.1
COVID-19	Dolnośląskie	0.0
Neonatal disorders	Dolnośląskie	0.5
Other CMMND	Dolnośląskie	0.1
Self-harm and interpersonal violence	Dolnośląskie	0.3
Transport injuries	Dolnośląskie	0.5
Unintentional injuries	Dolnośląskie	0.4
Ischemic heart disease	Kujawsko-Pomorskie	2.5
Stroke	Kujawsko-Pomorskie	1.5
Neoplasms	Kujawsko-Pomorskie	0.9
Chronic respiratory diseases	Kujawsko-Pomorskie	0.3
Digestive diseases	Kujawsko-Pomorskie	0.0
Diabetes and kidney diseases	Kujawsko-Pomorskie	0.1
Other NCD	Kujawsko-Pomorskie	0.6
Lower respiratory infections	Kujawsko-Pomorskie	-0.1
COVID-19	Kujawsko-Pomorskie	-0.1
Neonatal disorders	Kujawsko-Pomorskie	0.6
Other CMMND	Kujawsko-Pomorskie	0.1
Self-harm and interpersonal violence	Kujawsko-Pomorskie	0.1
Transport injuries	Kujawsko-Pomorskie	0.5
Unintentional injuries	Kujawsko-Pomorskie	0.2
Ischemic heart disease	Lubelskie	1.6
Stroke	Lubelskie	1.9
Neoplasms	Lubelskie	0.8

Chronic respiratory diseases	Lubelskie	0.4
Digestive diseases	Lubelskie	0.0
Diabetes and kidney diseases	Lubelskie	0.1
Other NCD	Lubelskie	0.7
Lower respiratory infections	Lubelskie	-0.1
COVID-19	Lubelskie	-0.1
Neonatal disorders	Lubelskie	0.5
Other CMMND	Lubelskie	0.1
Self-harm and interpersonal violence	Lubelskie	0.1
Transport injuries	Lubelskie	0.4
Unintentional injuries	Lubelskie	0.2
Ischemic heart disease	Lubuskie	1.8
Stroke	Lubuskie	1.6
Neoplasms	Lubuskie	1.1
Chronic respiratory diseases	Lubuskie	0.3
Digestive diseases	Lubuskie	0.0
Diabetes and kidney diseases	Lubuskie	0.1
Other NCD	Lubuskie	0.8
Lower respiratory infections	Lubuskie	-0.1
COVID-19	Lubuskie	-0.1
Neonatal disorders	Lubuskie	0.5
Other CMMND	Lubuskie	0.1
Self-harm and interpersonal violence	Lubuskie	0.2
Transport injuries	Lubuskie	0.5
Unintentional injuries	Lubuskie	0.3
Ischemic heart disease	Łódzkie	2.4
Stroke	Łódzkie	1.7
Neoplasms	Łódzkie	0.7
Chronic respiratory diseases	Łódzkie	0.3
Digestive diseases	Łódzkie	-0.1
Diabetes and kidney diseases	Łódzkie	0.1
Other NCD	Łódzkie	0.8
Lower respiratory infections	Łódzkie	-0.2
COVID-19	Łódzkie	-0.1
Neonatal disorders	Łódzkie	0.5
Other CMMND	Łódzkie	0.1
Self-harm and interpersonal violence	Łódzkie	0.1
Transport injuries	Łódzkie	0.5
Unintentional injuries	Łódzkie	0.2
Ischemic heart disease	Małopolskie	2.4
Stroke	Małopolskie	1.6
Neoplasms	Małopolskie	1.0
Chronic respiratory diseases	Małopolskie	0.3
Digestive diseases	Małopolskie	0.0

Diabetes and kidney diseases	Małopolskie	0.1
Other NCD	Małopolskie	0.6
Lower respiratory infections	Małopolskie	-0.1
COVID-19	Małopolskie	-0.1
Neonatal disorders	Małopolskie	0.5
Other CMMND	Małopolskie	0.1
Self-harm and interpersonal violence	Małopolskie	0.2
Transport injuries	Małopolskie	0.5
Unintentional injuries	Małopolskie	0.3
Ischemic heart disease	Mazowieckie	2.8
Stroke	Mazowieckie	1.8
Neoplasms	Mazowieckie	1.1
Chronic respiratory diseases	Mazowieckie	0.3
Digestive diseases	Mazowieckie	0.0
Diabetes and kidney diseases	Mazowieckie	0.1
Other NCD	Mazowieckie	0.7
Lower respiratory infections	Mazowieckie	-0.2
COVID-19	Mazowieckie	-0.1
Neonatal disorders	Mazowieckie	0.5
Other CMMND	Mazowieckie	0.1
Self-harm and interpersonal violence	Mazowieckie	0.2
Transport injuries	Mazowieckie	0.6
Unintentional injuries	Mazowieckie	0.3
Ischemic heart disease	Opolskie	2.4
Stroke	Opolskie	2.0
Neoplasms	Opolskie	0.7
Chronic respiratory diseases	Opolskie	0.3
Digestive diseases	Opolskie	0.0
Diabetes and kidney diseases	Opolskie	0.1
Other NCD	Opolskie	0.4
Lower respiratory infections	Opolskie	-0.1
COVID-19	Opolskie	0.0
Neonatal disorders	Opolskie	0.5
Other CMMND	Opolskie	0.1
Self-harm and interpersonal violence	Opolskie	0.1
Transport injuries	Opolskie	0.4
Unintentional injuries	Opolskie	0.3
Ischemic heart disease	Podkarpackie	2.7
Stroke	Podkarpackie	1.6
Neoplasms	Podkarpackie	0.9
Chronic respiratory diseases	Podkarpackie	0.3
Digestive diseases	Podkarpackie	0.0
Diabetes and kidney diseases	Podkarpackie	0.1
Other NCD	Podkarpackie	0.5

Lower respiratory infections	Podkarpackie	-0.1
COVID-19	Podkarpackie	0.0
Neonatal disorders	Podkarpackie	0.5
Other CMMND	Podkarpackie	0.1
Self-harm and interpersonal violence	Podkarpackie	0.1
Transport injuries	Podkarpackie	0.5
Unintentional injuries	Podkarpackie	0.2
Ischemic heart disease	Podlaskie	1.6
Stroke	Podlaskie	1.5
Neoplasms	Podlaskie	0.8
Chronic respiratory diseases	Podlaskie	0.3
Digestive diseases	Podlaskie	0.0
Diabetes and kidney diseases	Podlaskie	0.1
Other NCD	Podlaskie	0.8
Lower respiratory infections	Podlaskie	-0.1
COVID-19	Podlaskie	-0.1
Neonatal disorders	Podlaskie	0.5
Other CMMND	Podlaskie	0.1
Self-harm and interpersonal violence	Podlaskie	0.2
Transport injuries	Podlaskie	0.5
Unintentional injuries	Podlaskie	0.4
Ischemic heart disease	Pomorskie	2.6
Stroke	Pomorskie	1.6
Neoplasms	Pomorskie	1.7
Chronic respiratory diseases	Pomorskie	0.3
Digestive diseases	Pomorskie	0.0
Diabetes and kidney diseases	Pomorskie	0.2
Other NCD	Pomorskie	0.5
Lower respiratory infections	Pomorskie	0.0
COVID-19	Pomorskie	-0.1
Neonatal disorders	Pomorskie	0.5
Other CMMND	Pomorskie	0.1
Self-harm and interpersonal violence	Pomorskie	0.2
Transport injuries	Pomorskie	0.5
Unintentional injuries	Pomorskie	0.3
Ischemic heart disease	Śląskie	3.0
Stroke	Śląskie	1.5
Neoplasms	Śląskie	0.9
Chronic respiratory diseases	Śląskie	0.3
Digestive diseases	Śląskie	-0.1
Diabetes and kidney diseases	Śląskie	0.2
Other NCD	Śląskie	0.6
Lower respiratory infections	Śląskie	-0.1
COVID-19	Śląskie	-0.1

Neonatal disorders	Śląskie	0.5
Other CMMND	Śląskie	0.1
Self-harm and interpersonal violence	Śląskie	0.2
Transport injuries	Śląskie	0.4
Unintentional injuries	Śląskie	0.3
Ischemic heart disease	Świętokrzyskie	2.2
Stroke	Świętokrzyskie	1.2
Neoplasms	Świętokrzyskie	-0.1
Chronic respiratory diseases	Świętokrzyskie	0.4
Digestive diseases	Świętokrzyskie	-0.2
Diabetes and kidney diseases	Świętokrzyskie	0.1
Other NCD	Świętokrzyskie	0.2
Lower respiratory infections	Świętokrzyskie	-0.1
COVID-19	Świętokrzyskie	-0.1
Neonatal disorders	Świętokrzyskie	0.5
Other CMMND	Świętokrzyskie	0.1
Self-harm and interpersonal violence	Świętokrzyskie	0.0
Transport injuries	Świętokrzyskie	0.5
Unintentional injuries	Świętokrzyskie	0.2
Ischemic heart disease	Warmińsko-Mazurskie	2.6
Stroke	Warmińsko-Mazurskie	1.5
Neoplasms	Warmińsko-Mazurskie	1.2
Chronic respiratory diseases	Warmińsko-Mazurskie	0.4
Digestive diseases	Warmińsko-Mazurskie	-0.1
Diabetes and kidney diseases	Warmińsko-Mazurskie	0.1
Other NCD	Warmińsko-Mazurskie	0.5
Lower respiratory infections	Warmińsko-Mazurskie	-0.2
COVID-19	Warmińsko-Mazurskie	-0.1
Neonatal disorders	Warmińsko-Mazurskie	0.4
Other CMMND	Warmińsko-Mazurskie	0.1
Self-harm and interpersonal violence	Warmińsko-Mazurskie	0.2
Transport injuries	Warmińsko-Mazurskie	0.6
Unintentional injuries	Warmińsko-Mazurskie	0.4
Ischemic heart disease	Wielkopolskie	3.1
Stroke	Wielkopolskie	1.3
Neoplasms	Wielkopolskie	0.8
Chronic respiratory diseases	Wielkopolskie	0.3
Digestive diseases	Wielkopolskie	0.0
Diabetes and kidney diseases	Wielkopolskie	0.2
Other NCD	Wielkopolskie	0.7
Lower respiratory infections	Wielkopolskie	-0.2
COVID-19	Wielkopolskie	0.0
Neonatal disorders	Wielkopolskie	0.5
Other CMMND	Wielkopolskie	0.1

Self-harm and interpersonal violence	Wielkopolskie	0.1
Transport injuries	Wielkopolskie	0.4
Unintentional injuries	Wielkopolskie	0.2
Ischemic heart disease	Zachodniopomorskie	2.7
Stroke	Zachodniopomorskie	1.4
Neoplasms	Zachodniopomorskie	1.0
Chronic respiratory diseases	Zachodniopomorskie	0.3
Digestive diseases	Zachodniopomorskie	-0.1
Diabetes and kidney diseases	Zachodniopomorskie	0.1
Other NCD	Zachodniopomorskie	0.3
Lower respiratory infections	Zachodniopomorskie	-0.1
COVID-19	Zachodniopomorskie	-0.1
Neonatal disorders	Zachodniopomorskie	0.6
Other CMMND	Zachodniopomorskie	0.1
Self-harm and interpersonal violence	Zachodniopomorskie	0.2
Transport injuries	Zachodniopomorskie	0.5
Unintentional injuries	Zachodniopomorskie	0.3

Table S4. Gather checklist

Item #	Checklist item	Reporting location
Objectives and funding		
1	Define the indicator(s), populations (including age, sex, and geographic entities), and time period(s) for which estimates were made.	Main text methods overview
2	List the funding sources for the work.	Main text acknowledgement section
Data Inputs		
For all data inputs from multiple sources that are synthesized as part of the study:		
3	Describe how the data were identified and how the data were accessed.	Main text methods section, paragraph 2, "Data sources and processing"
4	Specify the inclusion and exclusion criteria. Identify all ad-hoc exclusions.	Main text methods section, paragraph 2, "Data sources and processing"
5	Provide information on all included data sources and their main characteristics. For each data source used, report reference information or contact name/institution, population represented, data collection method, year(s) of data collection, sex and age range, diagnostic criteria or measurement method, and sample size, as relevant.	Main text methods section, paragraph 2, "Data sources and processing"; citations also given on the GHDx (https://ghdx.healthdata.org/gbd-2023/sources)
6	Identify and describe any categories of input data that have potentially important biases (e.g., based on characteristics listed in item 5).	Main text methods section, paragraph 5, "Estimating the fatal and non-fatal burden of diseases and injuries"
For data inputs that contribute to the analysis but were not synthesized as part of the study:		
7	Describe and give sources for any other data inputs.	N/A
For all data inputs:		
8	Provide all data inputs in a file format from which data can be efficiently extracted (e.g., a spreadsheet rather than a PDF), including all relevant meta-data listed in item 5. For any data inputs that cannot be shared because of ethical or legal reasons, such as third-party ownership, provide a contact name or the name of the institution that retains the right to the data.	Data inputs in excel format available on the GHDx (https://ghdx.healthdata.org/gbd-2023/sources)
Data analysis		
9	Provide a conceptual overview of the data analysis method. A diagram may be helpful.	Main text methods overview, paragraph 1
10	Provide a detailed description of all steps of the analysis, including mathematical formulae. This description should cover, as relevant, data cleaning, data pre-processing, data adjustments and weighting of data sources, and mathematical or statistical model(s).	Main text methods
11	Describe how candidate models were evaluated and how the final model(s) were selected.	Main text methods
12	Provide the results of an evaluation of model performance, if done, as well as the results of any relevant sensitivity analysis.	Main text methods
13	Describe methods for calculating uncertainty of the estimates. State which sources of uncertainty were, and were not, accounted for in the uncertainty analysis.	Main text methods
14	State how analytic or statistical source code used to generate estimates can be accessed.	The statistical code used in GBD 2023 is available: https://ghdx.healthdata.org/gbd-2023/code

		(note: the link will be made public upon publication)
Results and Discussion		
15	Provide published estimates in a file format from which data can be efficiently extracted.	GBD 2023 results tool: https://vizhub.healthdata.org/gbd-results (note: this tool is not yet live but will be made public upon publication)
16	Report a quantitative measure of the uncertainty of the estimates (e.g. uncertainty intervals).	UIs given for all findings, including in the text, figures, and tables in the main text and all supplementary materials; online viz tools (see information above)
17	Interpret results in light of existing evidence. If updating a previous set of estimates, describe the reasons for changes in estimates.	Main text discussion
18	Discuss limitations of the estimates. Include a discussion of any modelling assumptions or data limitations that affect interpretation of the estimates.	Main text discussion, limitations section

Authors’ Contributions

Providing data or critical feedback on data sources

Ayman Ahmed, Abid Ali, Mohammad Al-Wardat, Maciej Banach, Mohammad-Mahdi Bastan, Sonu Bhaskar, Vijay Kumar Chattu, Temitope Cyrus Ekundayo, Chadi Eltaha, Simon I Hay, M. Azhar Hussain, Jacek Jerzy Jozwiak, Adnan Kisa, Zbigniew J Król, Munjae Lee, Maciej Mi kowski, Ali H Mokdad, Christopher J L Murray, Mohsen Naghavi, Van Thanh Nguyen, Fred Nugen, Samuel M Ostroff, Mahesh P A, Maja Pasovic, Shakthi Kumaran Ramasamy, Cameron John Sabet, Mehdi Safari, Mahabalesh Shetty, Aminu Shittu, Chandan Kumar Swain, Lukasz Szarpak, Roman Topor-Madry, Anna Weszka, Marcin W Wojewodzic, Bogdan Wojtyniak

Developing methods or computational machinery

Simon I Hay, Ali H Mokdad, Christopher J L Murray, Mohsen Naghavi, Maja Pasovic, Roman Topor-Madry

Providing critical feedback on methods or results

Ayman Ahmed, Abid Ali, Waad Ali, Mohammed A Alsabri, Mohammad Al-Wardat, Shahkaar Aziz, Maciej Banach, Mohammad-Mahdi Bastan, Sonu Bhaskar, Vijay Kumar Chattu, Sheng-Chia Chung, Kuldeep Dhama, Arkadiusz Marian Dziedzic, Temitope Cyrus Ekundayo, Chadi Eltaha, Patrick Fazeli, Xiang Gao, Miglas Welay Gebregergis, Simon I Hay, Chengxi Hu, M. Azhar Hussain, Jacek Jerzy Jozwiak, Adnan Kisa, Zbigniew J Król, Munjae Lee, Adam Maciejczyk, Hadush Negash Meles, Tomasz Miazgowski, Agnieszka Micek, Irmina Maria Michalek, Maciej Mi kowski, Ali H Mokdad, Christopher J L Murray, Mohsen Naghavi, Van Thanh Nguyen, Fred Nugen, Michal Ordak, Samuel M Ostroff, Mahesh P A, Maja Pasovic, Shakthi Kumaran Ramasamy, Elrashdy Redwan, Cameron John Sabet, Mohammad Reza Saeb, Mehdi Safari, Jennifer Saulam, Mohammad Ali Shamshirgaran, Aminu Shittu, Emmanuel Edwar Siddig, Chandan Kumar Swain, Lukasz Szarpak, Roman Topor-Madry, Muhammad Waqas, Marcin W Wojewodzic, Bogdan Wojtyniak

Drafting the work or revising it critically for important intellectual content

Ayman Ahmed, Mohammed Ahmed Akkaif, Abid Ali, Waad Ali, Mohammad Al-Wardat, Razman Arabzadeh Bahri, Maciej Banach, Mohammad-Mahdi Bastan, Maryam Beiranvand, Sonu Bhaskar, Catherine Bisignano, Vijay Kumar Chattu, Nicole Davis Weaver, Arkadiusz Marian Dziedzic, Chadi Eltaha, Nuno Ferreira, Xiang Gao, Miglas Welay Gebregergis, Snigdha Gulati, Simon I Hay, Chengxi Hu, M. Azhar Hussain, Jacek Jerzy Jozwiak, Adnan Kisa, Zbigniew J Król, Adam Maciejczyk, Hadush Negash Meles, Tomasz Miazgowski, Irmina Maria Michalek, Maciej Mi kowski, Ali H Mokdad, Christopher J L Murray, Mohsen Naghavi, Van Thanh Nguyen, Fred Nugen, Michal Ordak, Samuel M Ostroff, Mahesh P A, Maja Pasovic, Shakthi Kumaran Ramasamy, Elrashdy Redwan, Cameron John Sabet, Mehdi Safari, Mahabalesh Shetty, Aminu Shittu, Emmanuel Edwar Siddig, Chandan Kumar Swain, Lukasz Szarpak, Roman Topor-Madry, Anna Weszka, Marcin W Wojewodzic, Bogdan Wojtyniak

Managing the estimation or publications process

Simon I Hay, Johan Månsson, Ali H Mokdad, Christopher J L Murray, Mohsen Naghavi, Samuel M Ostroff, Maja Pasovic
