# Supplementary material for: The burden of diseases, injuries, and risk factors by voivodship in Poland, 1990–2023: a systematic analysis for the Global Burden of Disease Study 2023
Source: Lancet Reg Health Eur. 2025 Sep 3;58:101431. doi: 10.1016/j.lanepe.2025.101431 (PMC12624802; doi:10.1016/j.lanepe.2025.101431)
Supplement: Poland Summary [file mmc2.docx]

***This translation in Polish was submitted by the authors and we reproduce it as supplied. It has not been peer reviewed. Our editorial processes have only been applied to the original abstract in English, which should serve as reference for this manuscript.***

Tło
Od 1990 roku głębokie przemiany polityczne i gospodarcze w Polsce istotnie wpłynęły na system ochrony zdrowia i kształtowanie polityki zdrowotnej. Naszym celem było pogłębione pokazanie zmian, które zaszły w stanie zdrowia ludności Polski oraz w regionach pozwalające na lepsze ich zrozumienie przy wykorzystaniu danych z badania Global Burden of Diseases, Injuries, and Risk Factors Study (GBD) 2023.

Metody
GBD 2023 ocenia wyniki zdrowotne dla 375 chorób i urazów, 292 przyczyn zgonów oraz 88 czynników ryzyka w 204 krajach i terytoriach. W niniejszej pracy analizowaliśmy umieralność, oczekiwaną długość życia, obciążenie związane z czynnikami ryzyka, lata przeżyte z niepełnosprawnością (YLD), lata życia utracone z powodu przedwczesnego zgonu (YLL) oraz lata życia skorygowane o niepełnosprawność (DALY - łączna miara obciążenia chorobowego będąca sumą YLL i YLD). Uwzględniliśmy również dekompozycję zmian długości życia według przyczyn zgonów w Polsce i województwach w latach 1990-2023 na tle wybranych krajów europejskich. Oszacowania wskaźników zdrowia przedstawiono na poziomie krajowym i regionalnym według wieku (25 grup wiekowych), płci (mężczyźni, kobiety i łącznie) i roku. Ostateczne wartości punktowe przedstawiono z 95% przedziałami niepewności (UI), wyznaczonymi jako 2,5 i 97,5 percentyl z rozkładu 250 iteracji dla każdej miary.

Wyniki
W latach 1990-2023 oczekiwana długość życia w Polsce wzrosła z 71,2 (71,1-71,3) do 78,6 (78,5-78,7) lat. W tym okresie oczekiwana długość życia mężczyzn zwiększyła się bardziej niż kobiet: u mężczyzn z 66,8 (66,7-66,9) do 74,9 (74,8-75,0) lat, a u kobiet z 75,7 (75,6-75,8) do 82,2 (82,1-82,3) lat. Największy wpływ na poprawę miała redukcja liczby zgonów z powodu choroby niedokrwiennej serca i udaru: w 1990 roku odnotowano odpowiednio 109 240 (104 220-114 830) i 68 270 (63 690-73 590) zgonów, a w 2023 roku 85 360 (76 780-90 770) i 39 470 (35 010-42 940). Województwem o najwyższym standaryzowanym względem wieku wskaźniku DALY na 100 000 mieszkańców w 2023 roku było łódzkie (25 607 [22 602-29 217]), a najniższym - małopolskie (22 113 [19 290-25 418]). Wskaźnik DALY standaryzowany względem wieku spadł o 33,9% (-30,7 do -36,8) między 1990 a 2023 rokiem. Palenie tytoniu i nadciśnienie tętnicze pozostawały głównymi czynnikami ryzyka utraty zdrowia przez cały badany okres. Największy wzrost wskaźników DALY zaobserwowano w przypadku spożywania alkoholu (wzrost o 35,2% [3,5-65,1]). DALY przypisywane czynnikom ryzyka zmniejszyły się m.in. dla nadciśnienia (1990: 5723,8 [4759,8-6578,3]; 2023: 2053,7 [1657,2-2362,4]) i wysokiego BMI (1990: 2226,4 [966,8-3512,2]; 2023: 1923,4 [839,0-2907,8]).

Interpretacja
Od 1990 roku oczekiwana długość życia w Polsce wzrosła zarówno u kobiet, jak i u mężczyzn - przy czym to mężczyźni zyskali więcej dodatkowych lat życia. W 2023 roku wskaźnik ten był wyższy niż w Europie Środkowo-Wschodniej, ale niższy niż w Europie Zachodniej. Wyniki badania GBD 2023 ukazują istotne różnice w obciążeniu zarówno chorobami jak i czynnikami ryzyka ludności Polski w porównaniu z mieszkańcami krajów Europy Zachodniej oraz między mieszkańcami poszczególnych województw i sugerują, że poprawę stanu zdrowia w Polsce oraz zmniejszenie jego zróżnicowania między województwami w dużym stopniu można osiągnąć koncentrując działania na modyfikowalnych czynnikach ryzyka, zwłaszcza paleniu tytoniu i spożyciu alkoholu.
